# Technological and Biotechnological Processes To Enhance the Bioavailability of Dietary (Poly)phenols in Humans

**DOI:** 10.1021/acs.jafc.1c07198

**Published:** 2022-02-14

**Authors:** Franck Polia, Marta Pastor-Belda, Alberto Martínez-Blázquez, Marie-Noelle Horcajada, Francisco A. Tomás-Barberán, Rocío García-Villalba

**Affiliations:** † Laboratory of Food & Health, Research Group on Quality, Safety and Bioactivity of Plant Foods, Centro de Edafología y Biología Aplicada del Segura−Consejo Superior de Investigaciones Científicas (CEBAS−CSIC), Campus de Espinardo 25, 30100 Murcia, Spain; ‡ Department of Analytical Chemistry, Faculty of Chemistry, Regional Campus of International Excellence “Campus Mare Nostrum”, University of Murcia, 30100 Murcia, Spain; § Nestlé Research, Innovation EPFL Park, 1015 Lausanne, Switzerland

**Keywords:** (poly)phenols, bioavailability, food processing, enzymatic hydrolysis, probiotics

## Abstract

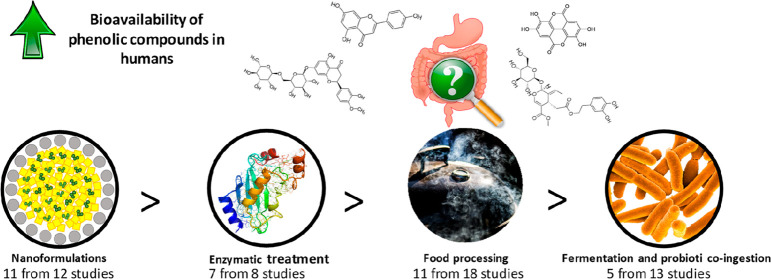

The health effects of (poly)phenols (PPs) depend upon their bioavailability that, in general, is very low and shows a high interindividual variability. The low bioavailability of PPs is mainly attributed to their low absorption in the upper gastrointestinal tract as a result of their low water solubility, their presence in foods as polymers or in glycosylated forms, and their tight bond to food matrices. Although many studies have investigated how technological and biotechnological processes affect the phenolic composition of fruits and vegetables, limited information exists regarding their effects on PP bioavailability in humans. In the present review, the effect of food processing (mechanical, thermal, and non-thermal treatments), oral-delivery nanoformulations, enzymatic hydrolysis, fermentation, co-administration with probiotics, and generation of postbiotics in PP bioavailability have been overviewed, focusing in the evidence provided in humans.

## 1 Introduction

Dietary (poly)phenols (PPs) have been associated with health benefits in many epidemiological studies.^1^ However, the demonstration of the biological effects in randomized controlled clinical trials has been relatively obscure as a result of the large interindividual variability observed.^2,3^ The health-promoting effects of phenolic compounds depend upon their bioaccessibility (fraction of an ingested compound that is available for absorption in the gut) and their consequent bioavailability (fraction of an ingested compound that reaches the systemic circulation and tissues to exert its biological action). Although PP bioavailability varies broadly, it is generally limited.^4,5^ This variation can be due to several factors, including the variability in the human transporters and phase I and phase II metabolism, the gut microbiota metabolism, and the dietary habits and food matrix effects, among others.^6−9^ The low bioavailability of phenolic compounds is closely linked to its poor bioaccessibility. They cannot be generally absorbed in the upper gastrointestinal tract because they are unable to cross the lipid barrier, influenced among other factors by their solubility in water. Besides, PPs often occur in foods as polymers or in glycosylated forms that hamper their absorption. They must suffer hydrolysis by the intestinal enzymes or the colonic microflora before absorption and often do not retain the original phenolic core structure, leading to simpler phenolic metabolites.^10^ Furthermore, a significant amount of phenolic compounds are bound tightly to food matrices through covalent bonds to cell wall structural components, which again hinder their absorption from the upper gastrointestinal tract.^4^

A key aspect of dietary PP research is to explore technological, biotechnological, and nutritional strategies that can increase PP efficacy, enhancing their bioavailability, and, therefore, help overcome the interindividual variability observed in clinical trials. A relevant research objective will be to find methods to increase the parent PP bioavailability or to facilitate their transformation into more bioavailable metabolites.

Nutritional strategies based on the interaction of PPs with other macronutrients (carbohydrates, lipids, and proteins), micronutrients (vitamins and minerals), and other molecules present in the human diet have been widely studied.^11,12^ Recent reviews highlighted the current knowledge on the influence of the food matrix on PP bioaccessibility and bioavailability.^12,13^ Despite some contradictory results, Kamiloglu et al. concluded that the presence of proteins, dietary fiber, and minerals might reduce the bioavailability of several flavonoids.^13^ In contrast, lipids, carbohydrates, vitamins, carotenoids, and other flavonoids are likely to improve flavonoid bioavailability. However, it is not easy to obtain general conclusions, and every food matrix and compound should be evaluated individually.

Technological and biotechnological processes can induce chemical or physical modifications in food or individual PPs to enhance their bioaccessibility and bioavailability. These changes include (1) food structure changes that lead to the release of phenolic compounds from the matrix, (2) formulations based on nanoparticles that protect phenolic compounds until they are absorbed, (3) chemical and enzymatic modifications into more bioavailable forms (i.e., hydrolysis of PPs into other phenolic compounds with improved bioavailability), and (4) conversion in postbiotic metabolites by gut microbial strains to produce ingredients that could be generally recognized as safe (GRAS) and added to food formulations as ingredients.

There are many studies on the effects of different food processing technologies on PP content and antioxidant activity.^14,15^ On the basis of the results of the phenolic content, authors have hypothesized the effects expected in bioavailability. However, sometimes bioavailability studies do not support the changes observed in PP profiles. More recently, the interest has focused on the evaluation of bioaccessibility and bioavailability. Bioaccessibility is assessed by *in vitro* methods that simulate digestion conditions to estimate the amount of compounds available for intestinal absorption, whereas bioavailability is generally measured using *in vivo* analysis of the metabolites in blood [area under the curve (AUC) in pharmacokinetic studies] and/or urine (total amount excreted). The assessment of bioaccessibility of phenolic compounds using *in vitro* gastrointestinal digestion models is a standard tool in food technology research.^16^ Many *in vitro* studies compiled in different reviews aimed to understand the effect of food processing on bioactive compound bioaccessibility, especially for carotenoids.^17−20^ However, data from *in vitro* studies that do not consider bioavailability and metabolism *in vivo* should be taken with caution. *In vivo* studies that evaluate the effect of processing on the bioavailability of phenolic compounds are still scarce.

**Figure 1:**
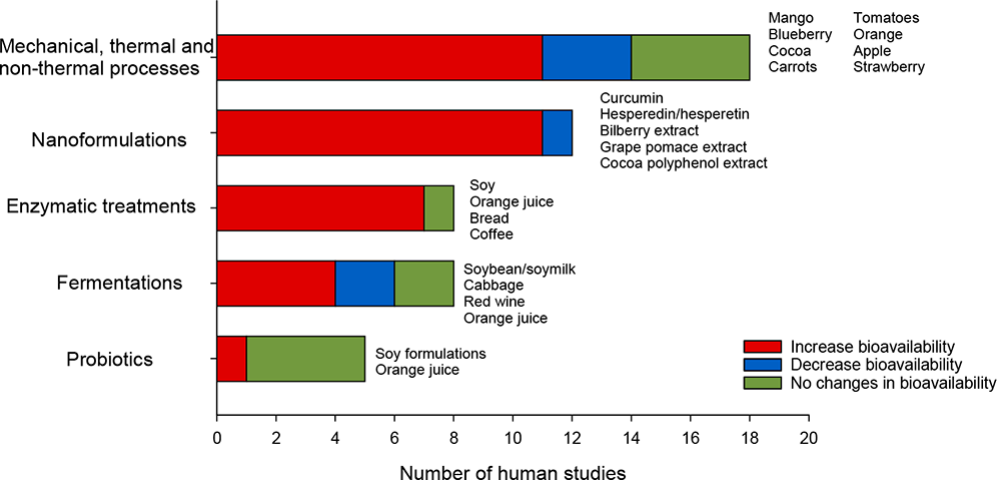
Summary of the human studies that evaluate the effect of different technological and biotechnological treatments on polyphenol bioavailability.

In the present review, the effect of different technological and biotechnological processes on PP bioavailability was studied, focusing on the evidence provided by human studies. The search was focused on all human studies published along the time about this issue in important databases, such as Web of Science, SCOPUS, and PubMed. Overall, 51 articles were reviewed, and a summary of the number of human studies evaluated in this review for each type of treatment can be observed in Figure 1.

## 2 Technological Processes

This section includes food processing technologies that could modify the food matrix in which PPs are entrapped, to facilitate their release and, therefore, their bioaccessibility. Innovative nanoformulations that could increase the stability and solubility of PPs, resulting in improved bioavailability, are also included here.

### 2.1 Food Processing

Many studies have investigated how technological processes affect the phenolic composition of fruits and vegetables.^15^ Still, limited information exists regarding the effects of processing on the PP bioavailability in humans. This section considered the impact of food processing, mainly mechanical, thermal, and non-thermal treatments, on the bioavailability of different phenolic compound families, focusing on human studies (Table 1). The initial goal of most studies was not to improve bioavailability but to study the influence of the technological process applied.

**Table 1:** Human Studies of the Effect of Food Processing on Bioavailability of PPs

matrix	food processing	volunteers	results after processing	reference
mango	juicing	healthy men (*n* = 12)	↑ AUC in plasma for chlorogenic acid (4.4-fold) and ferulic acid (2.4-fold) with *T*_max_ slightly shorter	(21)
			↑ urinary excretion at 0–4 h of *p*-coumaric acid (10-fold) and ferulic acid (3.6-fold)^a^	
blueberry	juicing	healthy (*n* = 9)	↓ intensity of 15% of all ions detected in non-targeted analysis in plasma and urine samples, including conjugated phenolic metabolites, such as ferulic and caffeic acids	(22)
cocoa powder	alkalinization	healthy (*n* = 12)	alkalinization induces an epimerization of (−)-epicatechin to (−)-catechin, a typical stereoisomer less bioavailable than the native epicatechin (plasma)	(23)
purple carrot	microwave cooked	healthy (*n* = 12)	↑ percent recovered in plasma (1.3-fold) and urine (1.4-fold) of non-acylated anthocyanins	(25)
			no effect on acylated anthocyanins	
cherry tomato	domestic cooking	healthy (*n* = 5)	↑ plasma concentrations of naringenin (from non-detected to 0.06 μmol/L) and chlorogenic acid (around 3-fold)^a^	(26)
tomato	boiling and crushing (tomato sauce production)	healthy (*n* = 8)	↑ AUC in plasma (11-fold) and urinary excretion (8.3-fold) of naringenin glucuronide	(27)
tomato	boiling and crushing (tomato sauce production)	healthy (*n* = 40)	↑ *C*_max_ in plasma of naringenin (1.4-fold), naringenin glucuronide (2.8-fold); no significant changes in AUC because of variability; ↑ AUC in plasma of quercetin (1.5-fold); no effect on gut microbial metabolites	(28)
blueberry	cooking, proving, and baking (blueberry-containing baked products); comparison to blueberry drink	healthy men (*n* = 10)	AUC in plasma of total (poly)phenols was similar for both treatments	(29)
			↑ AUC for hydroxyphenylacetic acid (2.5-fold), ferulic acid (1.5-fold), isoferulic acid (1.5-fold), and hydroxyhippuric acid (1.5-fold)	
			↓ AUC for hippuric acid (1.7-fold), benzoic acid (1.7-fold), salicylic acid (2.9-fold), and sinapic acid (2.6-fold)	
orange	juicing and pasteurization (pasteurized orange juice)	healthy (*n* = 12)	↑ bioavailability; despite the 2.4-fold higher doses of flavanones provided by the fresh fruit, no significant differences between both treatments were found in the urinary excretion of flavanones (hesperetin and naringenin) and their microbial metabolites	(30)
orange	juicing and pasteurization (commercial orange juice)	healthy (*n* = 20)	non-significant differences in urinary flavanone excretion	(31)
orange juice	pasteurization versus fresh hand-squeezed juice	healthy (*n* = 18)	non-significant differences in the relative urinary excretion of hesperetin and naringenin present in the soluble fraction	(32)
red-fleshed apple	freeze-dried apple, hot air-dried apple, and pasteurized apple purée	healthy women (*n* = 3)	↑ % excretion of total polyphenols in urine in pasteurized apple purée (3-fold) and hot air-dried apple (1.8-fold) compared to freeze-dried apple;^a^ no significant differences in the urinary excretion of anthocyanins	(33)
blackcurrant	drink made from a commercial highly processed blackcurrant syrup	healthy (*n* = 10)	processing reduced the content of anthocyanins but did not enhance the urinary yield of these compounds that was very low (<0.1%)	(34)
grape/blueberry	extrusion and pasteurization (smoothie versus juice)	healthy (*n* = 10)	no difference between juice and smoothie in plasma pharmacokinetics and urinary recoveries of the major anthocyanins	(35)
			↑ AUC in plasma (1.8-fold) of 3,4-dihydrobenzoic acid after ingestion of the juice compared to the smoothie	
apple	extrusion and pasteurization (smoothie versus juice)	healthy ileostomy (*n* = 10)	↑ recovery of total polyphenols (1.5-fold) in the ileostomy bag after smoothie intake compared to the juice, indicating more absorption of the compounds with the juice	(37)
strawberry	crushing and heated (strawberry purée)	healthy (*n* = 20)	no significant differences in the production and urinary excretion of ellagitannin gut microbial metabolites, urolithins	(38)
cocoa powder	fermentation, drying, and roasting (conventional versus unprocessed cocoa powder)	healthy (*n* = 6)	↓ epicatechin glucuronide in plasma (5-fold) and urinary excretion of different metabolites (2–12-fold), mainly methyl epicatechin sulfate	(39)
orange juices	HPP versus pasteurized and fresh hand-squeezed	healthy (*n* = 18)	↑ urinary excretion relative to soluble flavanones in the group of high flavanone excretors in HPP compared to pasteurized (2.1-fold) and fresh (1.7-fold)	(32)

a Data were obtained from graphics.

Most fruits are regularly processed by juicing or puréeing, techniques that could affect the PP bioavailability. However, evaluating just the juicing process is problematic because it is usually accompanied by an additional heat treatment (pasteurization) that will be discussed later. With regard to freshly prepared juices, it is expected that, although the process of juicing itself decreases the content of PPs, because most of them concentrate in the skin, the lower range of cell wall constituents and fiber in the juice could help to enhance the bioavailability. However, the scarce evidence from the literature is not conclusive. Quiros-Sauceda et al. suggested that processing mango flesh into fresh juice can increase the absorption in plasma and excretion in urine of some phenolic acids (chlorogenic, ferulic, and *p*-coumaric acids).^21^ It is hypothesized that phenolics are released from the fiber during juicing and could be more bioaccessible. On the contrary, Langer et al. observed that 15% of all ions detected using a non-targeted metabolomics profiling approach were significantly higher after whole blueberry intake than freshly prepared juices, including conjugated phenolic metabolites, such as ferulic and caffeic acids.^22^ However, this study presents the limitation that only the 2 h time point was used.

Another pretreatment studied is the alkalinization of the cocoa powder used in non-confectionery products to improve solubility and sensory properties. Plasma samples of 12 volunteers were analyzed 6 h after consuming a milk-based cocoa beverage prepared from either non-alkalinized or alkalinized cocoa powder. Alkalinization of cocoa powder induced the epimerization of (−)-epicatechin to (−)-catechin, a stereoisomer that is less bioavailable than the native epicatechin.^23^ A plasma analysis for up to 24 h with a larger sample size and considering other metabolites are necessary to obtain valid conclusions.

The effect of extrusion of sorghum seeds on catechin bioavailability was also studied in a randomized crossover design. Weaning pigs consumed either a sorghum mixture or the sorghum mixture extrudate. Extrusion improved plasma levels and urinary excretion of catechins, suggesting the release of proanthocyanidins and catechins from their binding with macromolecules and possible depolymerization of polymeric proanthocyanidins in the extruded sorghum.^24^

Thermal treatments are commonly applied in food processing in both domestic (boiling, frying, steaming, baking, stewing, roasting, and toasting) and industrial (drying, pasteurization, and sterilization) settings. Although the changes in PPs during thermal processing and their impact on their *in vitro* bioaccessibility have been widely reported,^15,19,20^ its effects on human PP bioavailability has been less studied.

In general, the bioavailability in a thermally treated food product depends upon a balance between the compounds degraded during processing and those released and better absorbed thanks to the changes induced in the matrix. Thermal processing also inactivates PP-degrading enzymes as polyphenol oxidase and, therefore, could maintain PPs better than a non-thermally processed food. The extent to which temperature affects phenolic compounds depends upon the matrix and PP chemical properties.^19^

Human studies about the effect of thermal processing on bioavailability are shown in Table 1. As mentioned above, in some studies, the thermal treatment is accompanied by other pretreatments, mainly mechanical, such as crushing, juicing, or puréeing.

The effect of cooking on the bioavailability of acylated and non-acylated anthocyanins was evaluated in a clinical study, in which volunteers consumed raw or microwave-cooked purple carrots. The cooking process only slightly improved the recovery in plasma and urine of non-acylated anthocyanins but did not affect acylated anthocyanins.^25^ For cherry tomatoes, home cooking increased the plasma concentrations of naringenin and chlorogenic acid (determined after enzymatic hydrolysis) in a crossover study, where volunteers had fresh and cooked tomatoes.^26^ The effects on PP bioavailability of the thermal and mechanical treatments applied during tomato sauce production have also been studied.^27^ The plasma concentration and urinary excretion of naringenin glucuronide were significantly higher after consuming tomato sauce compared to raw tomatoes. Because naringenin strongly interacts with insoluble polyesters, which are constituents of tomato peel fiber, mechanical and thermal processing could facilitate the release of the compound from the matrix and, thus, increase its bioaccessibility. The same authors performed another study with more volunteers and many phenolics.^28^ Processing tomatoes enhanced the bioavailability of flavanones (naringenin and naringenin glucuronide), flavonols (quercetin), and some hydroxycinnamic acids. A wide variety of gut microbial metabolites was also detected, although no significant differences were observed between samples, probably as a result of the variability among the volunteers.

The effect of processing (including cooking, proving, and baking) in the bioavailability of blueberry PPs was studied after consuming blueberry-containing baked products or an unprocessed blueberry drink containing the same amount of freeze-dried blueberry powder.^29^ Although the plasma AUC of total PPs was similar for both treatments, significant differences were observed in the levels of eight individual plasma metabolites. Hydroxyphenylacetic, ferulic, isoferulic, and hydroxyhippuric acids were higher in plasma after consuming the blueberry bun, whereas hippuric, benzoic, salicylic, and sinapic acids were higher after the blueberry drink intake. The increase in the chlorogenic acid content and the release of ferulic acid from the matrix in the processed bun may be responsible for increasing ferulic and isoferulic acids in plasma. The higher levels of hydroxyhippuric acid found after blueberry bun intake and the higher levels of benzoic and salicylic acids after the consumption of the drink suggested that processing facilitates the hydroxylation and glycination of the benzoic acid present in the freeze-dried blueberry. However, similar improvements in flow-mediated dilation were observed after consumption of both food products.

The effect of juicing or puréeing combined with thermal treatments on phenolic bioavailability has been widely reported for different PPs in different matrices, with contradictory results. Although this processing can occasionally increase the bioavailability of PPs, the conditions applied and the matrix have an essential influence on the results. A better flavanone bioavailability was observed after the consumption of pasteurized orange juice compared to fresh orange fruit. Despite the 2.4-fold higher dose of flavanones provided by the fresh fruit, no significant differences between both treatments were found in the urinary excretion of flavanones (hesperetin and naringenin) and their microbial metabolites [3-(3′-hydroxy-4′-methoxyphenyl) propionic acid, 3-(3′-hydroxyphenyl)hydracrylic acid, 4-hydroxyhippuric acid, and hippuric acid]. The authors hypothesized that this might be due to the saturation of transporters and the entrapment of flavonoids in the fiber-rich matrix of the fruit.^30^ On the contrary, in a randomized crossover trial comprising 20 subjects, Brett et al. reported no significant differences in urinary flavanone excretion after consuming 150 g of fresh oranges or 300 mL of commercial orange juice with similar amounts of hesperidin and narirutin.^31^ In another study, in which only a pasteurization treatment was applied, the relative urinary excretion of hesperetin and naringenin was similar to that of the fresh hand-squeezed juice.^32^

The processing effect on the PP bioavailability of red-fleshed apple was also studied in a human pilot study with three subjects consuming three apple products, freeze-dried apple, hot air-dried apple, and pasteurized apple purée, with a similar PP dose.^33^ Although freeze drying was the technology that preserved better PPs during processing, it was the product with the lowest bioavailability. In contrast, the pasteurized purée, with the highest losses during processing, showed the highest bioavailability. Therefore, apple processing could enhance PP bioavailability. No significant differences between treatments were observed in the urinary excretion of anthocyanins, the most affected by the processing, with even lower recoveries with the pasteurized purée. Similar results on anthocyanins were observed after blackcurrant processing.^34^ The blackcurrant drink made from a highly processed blackcurrant syrup showed lower anthocyanin content than in the original fruit. Processing did not improve the blackcurrant anthocyanin bioavailability that was, in all cases, very low (≤0.1%). In another study, the bioavailability of selected anthocyanins from a grape/blueberry juice (extruded and pasteurized) was compared to a smoothie, and no difference was found in plasma pharmacokinetics and recovery of the major anthocyanin species. Significantly higher concentrations of 3,4-dihydrobenzoic acid were detected after ingestion of the juice.^35^

In a recent study with minipigs challenged with a high-fat diet, the processing of apple into purée did not affect the bioavailability of flavan-3-ols. Still, a higher serum concentration of flavan-3-ol metabolites was obtained after a phenolic extract from apples was administered in comparison to raw apples.^36^ The reduced absorption with the matrix could originate from apple fiber, which may hamper bioaccessibility. In a human bioavailability study with ileostomy patients, significantly more PPs reached the ileostomy bags when an apple smoothie (containing 60% cloudy apple juice and 40% apple purée) was ingested versus an apple juice, indicating more absorption of the compounds with the juice.^37^ Smoothies are likely to have much higher PP contents than the respective juices but higher cell wall constituents because they are produced from whole fruits with lesser processing steps. Matrix components probably bind more PPs and, thus, reduce their bioavailability in the small intestine.

No significant differences in the production and excretion of ellagitannin gut microbial metabolites, urolithins, were found between the intake of fresh strawberries and a thermally processed strawberry purée (produced by microcrushing and slight thermal treatment) containing similar amounts of strawberries.^38^ Processing increased the amount of free ellagic acid, but neither thermal treatment nor the food matrix in which the ellagitannins are more accessible affected the transformation into urolithins by the gut microbiota.

Food processing could even negatively affect the PP content and bioavailability. This was observed in a study where an unprocessed cocoa powder (unfermented, non-roasted, and blanch-treated cocoa powder) showed better bioavailability than a conventional cocoa powder subjected to postharvest handling, fermentation, drying, and roasting.^39^ The content of epicatechin glucuronide in plasma was 5-fold higher upon consumption of the unprocessed cocoa than the conventional, and the urinary excretion of metabolites, mainly methyl epicatechin sulfate, was also higher (2–12-fold). This enhanced bioavailability seems to be primarily related to the higher flavonoid content (significantly enriched in monomer compounds) present in the new unprocessed cocoa powder.

Non-thermal processing technologies have been revealed as valuable tools to extend shelf life and preserve the nutritional and functional characteristics of fruit and vegetable products. However, there is scarce data on the effect of these emerging technologies on bioaccessibility and bioavailability of bioactive compounds. Only bioaccessibility studies with non-thermally processed foods have been developed, and little information about bioavailability is provided.^6,17,20^ The effect of high-pressure homogenization (HPH) processing on flavanone bioavailability in humans was assessed after consumption of fresh hand-squeezed, conventionally pasteurized, and HPH orange juices. Considering the urinary excretion relative to the soluble flavanones ingested, a significantly higher excretion was observed after HPH juice intake but only in the group of volunteers with a high flavanone excretion.^32^ The particle size, much smaller in the homogenized juice than in the pasteurized juice, leading to the microsuspension of the cloud, could improve solubility and accessibility of flavanones that would be better used by individuals stratified as high flavanone excretors.

### 2.2 Oral Delivery Nanoformulations

Oral delivery formulations based on nanotechnology have been developed to minimize the low stability, light sensitivity, low water solubility, and poor bioavailability of PPs. Several *in vitro* and *in vivo* studies with animal models compiled in different reviews have demonstrated that these formulations can improve the instability, bioavailability, and half-life of PPs, keeping their structural integrity and releasing them in a controlled manner.^40−43^Supplementary Table 1 of the Supporting Information summarizes some of the oral pharmacokinetic studies with animal models, mainly focused on curcumin, resveratrol, quercetin, and flavan-3-ols. Formulations based on nanoparticles, polymeric micelles, nanosuspensions, inclusion complexes with cyclodextrins, lipid-based nanoformulations, such as oil in water emulsions, self-nanoemulsifying drug delivery systems, phospholipid complexes (phytosomes), and solid lipid nanoparticles have demonstrated enhanced PP bioavailability.

Although results in animals are encouraging, how this formulation affects bioavailability in humans has been poorly studied. These formulations require sophisticated technologies that, in most cases, are not fully developed, and they are also unlikely to be cost-effective. Besides, the lack of toxicity data for the long-term human exposure to nanocarriers complicates the translation into food products. Only a few food-grade formulations, mainly focused on curcumin, have been studied in human clinical trials (Table 2).

**Table 2:** Human Clinical Trials Reporting Pharmacokinetic Parameters for Different PP Nanoformulations^a^

polyphenol	administration	volunteers	formulation	results (nanoformulations versus control)	reference
curcumin	capsules (2 g of CUR)	healthy (*n* = 11)	curcumin with turmeric essential oils BCM-95CG (Biocurcumax)	CUR: ↑ AUC (plasma) 7-fold with respect to the curcumin control and 6.4-fold with respect to the curcumin–lecithin–piperine formula	(44)
				longer retention time	
curcumin	capsules (130–195 mg of CUR)	osteosarcoma (*n* = 11) and healthy (*n* = 6)	solid lipid curcumin particles (SLCPs) by patent methodology (LONGVIDA and M3C-X)^b^	healthy: plasma *C*_max_, 22.43 ng/mL; *T*_max_, 2.4 h; AUC, 178.44 ng min/mL (no detection after unformulated extract administration)	(45)
				osteosarcoma: nonlinear dose dependency	
curcumin	dispersed in water (30 mg of CUR)	healthy (*n* = 14)	nanoparticle colloidal dispersion (THERACURMIN)^b^	CUR:^c^ ↑ AUC (plasma) 27.6-fold	(46)
				*T*_max_ is reduced from 6 to 1 h	
curcumin	capsules (376 mg of CUR)	healthy (*n* = 9)	Meriva, phospholipid complex with soybean lecithin (phytosome formulation) (Meriva)^b^	CUR:^c^ ↑ AUC 19-fold	(47)
				DMC:^c^ ↑ AUC 68-fold	
				BDMC:^c^ ↑ AUC 57-fold	
				total CURM:^c^ ↑ AUC 32-fold	
				*T*_max_ is reduced in all compounds	
				all determinations in plasma	
curcumin	bread enriched with encapsulated CUR^c^ alone (ECB) or in combination with piperine, genistein, and quercetin (ECBB) (1 g of CUR/100 g of bread portion)	healthy (*n* = 10)	microencapsulation with cellulose derivative and hydrogenated vegetable oil	total CURM: plasma, ↑ AUC 7.25-fold ECB and 4.58-fold ECBB; urine, ↑ AUC 1.2-fold ECB and ↓ AUC 1.5-fold ECBB	(48)
				CUR: plasma, ↓ AUC 7-fold ECB; ↑ AUC 2.8-fold ECBB; urine, ↑ AUC 7.8-fold ECB and 4.6-fold ECBB	
				DMC: plasma, ↑ AUC 3.4-fold ECB and 6.3-fold ECBB; urine, nd	
				BDMC: plasma, ↑ AUC 11.15-fold ECB and 2.2-fold ECBB; urine, nd	
				CURM-glucu: plasma, ↑ AUC 2.1-fold ECB and 3.6-fold ECBB; urine, ↑ AUC 1.3-fold ECB and 2.8-fold ECBB	
				phenolic acids: plasma, ↓ AUC 4-fold ECB; ↑ AUC 2.6-fold ECBB; urine, ↓ AUC 10-fold ECB; ↑ AUC 1.2-fold ECBB	
curcumin	mixed into woodruff syrup (500 mg of CUR)	healthy (*n* = 23)	micronized powder (MP) and liquid micelles (LM) with Tween 80	CUR:^c^ plasma, ↑ AUC 9-fold MP and 185-fold LM; urine, ↑ AUC 6-fold MP and 148-fold LM	(49)
				DMC:^c^ plasma, ↑ AUC 28-fold MP and 141-fold LM; urine, ↑ AUC 15-fold MP and 62-fold LM	
				BDMC:^c^ plasma, ↑ AUC 8.6-fold MP and 11.7-fold LM; urine, ↑ AUC 7-fold MP and 12-fold LM	
curcumin	capsules (376 mg of CUR)	healthy (*n* = 12)	comparison of three formulations: hydrophilic carrier, natural antioxidants, and cellulosic derivatives (CHC) versus phytosome (CP) versus oils of turmeric rhizome (CTR)	CUR:^c^ ↑ AUC 136.3-fold CHC, 12.7-fold CP, and 2.6-fold in CTR	(50)
				DMC:^c^ ↑ AUC 14.2-fold CHC, 7.3-fold CP, and 0.6-fold CTR	
				BDMC:^c^ ↑ AUC 5.3-fold CHC, 3.5-fold CP; and 1.3-fold CTR	
				THC:^c^ ↑ AUC 33.5-fold CHC, 8.3-fold CP, and 1.3-fold CTR	
				total CURM:^c^ ↑ AUC 45.9-fold CHC, 7.9-fold CP, and 1.3-fold CTR	
				all determinations in plasma	
curcumin	capsules (376 mg of CUR)	healthy (*n* = 12)	comparison of three formulations: γ-cyclodextrin inclusion complex (CC) versus phytosome (CP) versus CUR with oils of turmeric rhizome (CTR)	CUR:^c^ ↑ AUC 84-fold CC, 9-fold CP, and 1.7-fold CTR	(51)
				DMC:^c^ ↑ AUC 13.5-fold CC and 11-fold CP and CTR	
				BDMC:^c^ ↑ AUC 4.5-fold CC, 4.8-fold CP, and 1.4-fold CTR	
				total CURM:^c^ ↑ AUC 37.4-fold CC, 8.4-fold CP, and 1.2-fold CTR	
				all determinations in plasma	
hesperidin/hesperetin	added in non-flavanone-containing beverage (90 mg of hesperetin equivalents)	healthy (*n* = 18)	micronization (5.1 μm) (MHd and MHt) and coacervation–encapsulation with gum arabic (EHd)	MHd and EHd: ↑ urinary concentration of hesperetin equivalents (2.5-fold), especially in high and medium excretors	(52)
				MHt: ↑ urinary concentration of hesperetin equivalents (3.5-fold), especially in low excretors	
anthocyanins	encapsulated bilberry extract (10 g of ATS)	healthy (*n* = 5) and healthy ileostomics (*n* = 5)	nanoencapsulation with whey protein (WCP) or citrus pectin (CPC) by emulsification and thermal gelation	encapsulation did not strongly influence the total bioavailability of anthocyanins	(53)
				WCP: ↑ urinary concentration of ATs (1.7–2.2-fold) and degradation products (1.5-fold)	
				CPC: ↑ ileostomic concentration of ATs (1.2-fold)	
phenolic acids, stilbenes, flavan-3-ols, phenyl alcohols, and anthocyanins	red wine enriched with nanoencapsulated phenolic extract from grape pomace (1.3 g)	healthy (*n* = 12)	nanoencapsulation using zein nanoparticles and l-lysine (patented formulation)	↑ urine concentration of malvidin glucoside (1.8-fold), syringic acid sulfate (1.6-fold), glucuronide (1.4-fold), resveratrol sulfate (1.3-fold), and glucuronide (1.7-fold); no significant enhancement in plasma	(54)
flavanols and phenolic acids	cocoa nut creams enriched with free or microencapsulated cocoa polyphenol extract (385 μmol of flavanols and 13 μmol of phenolic acids)	healthy (*n* = 12)	microencapsulation with high-amylose maize starch	plasma: ↓ AUC_0–6 h_ (13.8-fold flavanols and 2-fold phenolic acids)	(55)
				urine: ↓ concentration (0–6 h) (29.8-fold flavanols and 12–8-fold phenolic acids)	
				feces: ↑ concentration (5.4-fold flavanols and 1.8-fold phenolic acids)	

a MHd, micronized hesperidin; MHt, micronized hesperetin; EHd, encapsulated hesperidin; ATs, anthocyanins; CUR, curcumin; DMC, demethoxy curcumin; BDMC, bisdemethocycurcumin; and THC, tetrahydroxycurcumin. When not indicated, AUC values refer to plasma.

b Curcumin from turmeric root extract.

c Concentration calculated after enzymatic hydrolysis.

A proprietary formulation [BCM-95CG (Biocurcumax)] combining curcuminoids with volatile oils of turmeric rhizome, which are usually eliminated during extraction, showed higher human bioavailability compared to standard curcumin (7-fold) and a curcumin–lecithin–piperine formula (6.4-fold).^44^ Besides, curcumin was absorbed early and retained longer from this new formulation. In another study with healthy volunteers, solid lipid curcumin particles (SLCPs) obtained by a patented methodology (LONGVIDA, M3C-X) showed curcumin plasma concentrations of 22.43 ng/mL after 2.4 h. At the same time, there was no curcumin found for unformulated curcumin.^45^ It is not clear if this enhanced bioavailability resulted from increased absorption or reduced conversion of free curcumin into conjugates because the samples were not treated with glucuronidase. The tolerance and dose plasma concentration of this formulation were evaluated in patients with osteosarcoma observing a nonlinear dose dependency that suggests complex absorption kinetics. Another curcumin formulation based on a nanoparticle colloidal dispersion prepared with gum ghatti and glycerine (THERACURMIN) demonstrated a higher bioavailability with shorter *T*_max_ and AUC_0–6 h_ values for total curcumin 27.6-fold higher compared to curcumin powder.^46^ This colloidal dispersion gave rise to a water-soluble and stable preparation of curcumin that enhanced gastrointestinal absorption. The inclusion of curcumin in a lipophilic matrix composed of curcumin/soybean lecithin/microcrystalline cellulose (1:2:2, Meriva) has been shown to increase the relative human absorption of curcumin and total curcuminoids by 19- and 32-fold, respectively. However, only phase II metabolites could be detected at low concentrations.^47^ Besides, this phospholipid formulation increased the absorption of demethoxylated curcuminoids much more than that of curcumin [68-fold for demethoxycurcumin (DMC) and 57-fold for bisdemethoxy curcumin (BDMC)]. In fact, DMC became the major plasma curcuminoid after consuming Meriva. Authors hypothesized that the hydrolytic stabilization of curcumin at intestinal pH might, in fact, translate into a significant curcumin load for the gut microbiota, known to be able to reductively demethoxylate dietary phenolics. Vitaglione et al. compared the bioavailability of curcumin from bread enriched with free curcumin (FCB), encapsulated curcumin in a cellulose derivative and hydrogenated vegetable oil coating (ECB), or encapsulated curcumin in combination with other bioactive compounds (piperine, quercetin, and genistein) (ECBB).^48^ Encapsulation protected curcuminoids from intestinal degradation, increasing the absorption of total curcuminoids (plasma AUC 7.25-fold higher) and decreasing the presence of degradation products (phenolic compounds) (plasma AUC 4-fold lower). The simultaneous administration of curcumin with other bioactive compounds improved the curcuminoid absorption less and increased their transformation into phenolic acids, such as ferulic and vanillic acids. This might be caused by the competitive absorption between curcumin and the other compounds at the intestinal mucosa level, leading to delayed curcumin absorption and a consequent increased degradation rate in the intestinal lumen. The micronized powder and particularly the liquid micellar formulation of curcumin significantly improved its oral bioavailability with AUC values in plasma for total curcumin 9- and 185-fold, respectively.^49^ Higher concentrations of DMC and BDMC were also observed. New formulations of curcumin, one with a combination of a hydrophilic carrier, cellulosic derivatives, and natural antioxidants (CHC)^50^ and the other with γ-cyclodextrin (CC),^51^ were compared to an unformulated extract and two commercially available formulations: phytosome formulation (CP) and a formulation with volatile oils of turmeric rhizome (CTR). The total concentration of curcuminoids in the new formulations was higher (45.9-fold in CHC and 37.4-fold in CC) compared to CP (7.9–8.4-fold) and CTR (1.2–1.3-fold).

Other formulations with flavanones (hesperidin) and anthocyanins were assayed in human studies. Hesperidin micronization (5.1 μm) and coacervation–encapsulation with gum arabic showed an increased urine excretion of hesperetin equivalents (2.5-fold), especially in individuals characterized as high and medium flavanone excretors. The bioavailability of micronized hesperetin was also increased, particularly for the low excretors (3.6-fold), showing the lack of appropriate microbiota in these volunteers to release hesperetin from hesperidin. With enhanced dispersion in water and decreased particle size, these formulations increased solubility in water and facilitated interaction with intestinal cells.^52^

In a recent study with healthy and ileostomy human volunteers, the encapsulation of bilberry extracts with either whey protein or citrus pectin did not strongly influence the bioavailability of anthocyanins.^53^ However, some modulatory effects could be observed. Whey protein encapsulation seems to modulate bioavailability with higher concentrations of anthocyanins and their degradation products in urine (although with contradictory results in plasma). Besides, citrus pectin nanoparticles seem to stabilize anthocyanins during the intestinal passage, finding higher concentrations of anthocyanins in the ileostomy effluents compared to non-encapsulated extracts. These last nanoparticles seem to modulate the formation of phloroglucinol aldehyde, the only degradation product with a high concentration in plasma and urine after administration of citrus pectin nanoparticles.

The impact of the nanoencapsulation in zein nanoparticles of a grape pomace phenolic extract was investigated after consumption of a dealcoholized red wine enriched with both non-encapsulated and nanoencapsulated extracts. Higher urinary excretion of malvidin-3-*O*-glucoside and the phase II conjugates (sulfate and glucuronide) of its microbial metabolite syringic acid reflected a slight enhancement of its bioavailability. The stability of anthocyanins was increased by encapsulation, which could ensure the steady and sustained release of anthocyanins in the colon. Resveratrol metabolites (sulfate and glucuronide conjugates) were also detected in higher concentrations with the nanoencapsulated formulation.^54^ Despite the slight increase observed in the bioavailability, the turbidity obtained after adding the encapsulated extract reduced the interest for using it in a final commercial functional wine. In a similar way, the human bioavailability of cocoa flavan-3-ols and phenolic acids was evaluated after consumption of cocoa nut cream enriched with a cocoa PP extract in free or encapsulated form with high amylose maize starch.^55^ Nanoencapsulation reduced the concentration of flavanols (epicatechin) and phenolic acids in plasma (13.8- and 2-fold, respectively) and urine (29.8- and 12.8-fold, respectively) in the first 6 h after ingestion and increased the concentration of these PPs in feces (5.4-fold flavanols and 1.8-fold phenolic acids). Encapsulation of cocoa PPs caused a reduced 24 h bioavailability of these compounds but allowed the delivery of flavanol monomers into the gut and the successive metabolism by the local microbiota. Therefore, from the nutritional point of view, encapsulated cocoa PPs may be considered a functional prebiotic ingredient.

## 3 Biotechnological Processes

Technologies involving living organisms and enzymes to enhance the bioavailability of PPs have gained special attention in the last years. These technologies are mainly applied to phenolics naturally found in food under a glycosylated, esterified, or polymerized form. In general, they show low bioavailability as a result of their high polarity or molecular weight, and they cannot be passively absorbed in the small intestine.^56^

### 3.1 Enzymatic Treatments

Enzymatic treatments before PP ingestion have been proposed to enhance their solubility and absorption or facilitate their interaction with gut microbes or intestinal enzymes. Differences in the phenolic composition of enzymatically hydrolyzed food have been widely reported,^57,58^ suggesting an improvement in bioavailability as a result of an increase of free components and a decrease of esterified and glycosidic compounds. However, there are few *in vivo* studies on the bioavailability of these hydrolyzed extracts (Table 3).

**Table 3:** Human Studies about the Influence of Enzymatic Treatments in the Bioavailability of PPs^a^

polyphenols (matrix)	enzymatic treatment	volunteers	results after hydrolysis versus control	reference
isoflavones (soy)	β-glucosidase (glycosides to aglycones)	healthy postmenopausal (*n* = 6)	no significant differences in plasma and urine	(59)
quercetin 3-*O*-β-rutinoside (pure)	enzymatic deglycosilation and subsequent α-oligoglucosylation (EMIQ)	healthy (*n* = 5)	↑ *C*_max_ in plasma of quercetin conjugates compared to the ingestion of Q3G (2.3-fold) and rutin (6.1-fold)^b^	(63)
hesperidin (orange juice)	rhamnosidase (hesperidin to hesperetin-7-glucoside)	healthy (*n* = 16)	↑ *C*_max_ (4-fold) and AUC (2-fold) in plasma of hesperetin	(66)
narirutin (orange juice)	rhamnosidase (narirutin to naringenin-7-glucoside)	healthy (*n* = 16)	↑ *C*_max_ (5.4-fold) and AUC in plasma (4-fold) of naringenin	(67)
			↑ urinary excretion (6.7-fold)	
ferulic acid and other phenolics (whole-meal bread)	xylanase, cellulose, α-amilase, β-glucanase, and feruloyl esterase (release phenolic from the food matrix)	healthy men (*n* = 8)	↑ AUC in plasma and urinary excretion for ferulic acid (2.7- and 2.2-fold) vanillic acid (1.8- and 1.6-fold), and 3,4-dimethoxybenzoic acid (1.8- and 1.9-fold)	(71)
			sinapic acid (nd and 2.4-fold)	
ferulic acid and other phenolics (white wheat bread fortified with bioprocessed rye bran)	feruloyl esterase (release phenolic from the food matrix) combined with yeast fermentation	healthy (*n* = 15)	↑ urinary excretion (4-fold) of ferulic acid	(72)
ferulic acid and other phenolics (high-fiber bread)	Ultraflo L (β-glucanase, xylanase, and feruloyl esterase activities)	healthy men (*n* = 19)	↑ *C*_max_ (3.2–6.4-fold) in plasma 2 h after consumption^b^	(74)
chlorogenic acid and other phenolics (coffee)	esterase of *Lactobacillus johnsonii* (phenolic acids from chlorogenic acids)	healthy (*n* = 12)	↑ AUC (3-fold) in plasma of all phenolic acid metabolites, and concentration was reached quickly (*T*_max_ changed from 9 to 11 to 1 h)	(76)

a EMIQ, enzymatically modified isoquercitrin; Q3G, quercetin-3-glucoside.

b Data were obtained from graphics.

Richelle et al. investigated whether the bioavailability of isoflavones could be enhanced by enzymatic hydrolysis with β-glucosidase of a non-fermented soy drink in postmenopausal women. The hydrolysis of isoflavone glucosides to aglycones before the consumption did not alter the plasma and urinary pharmacokinetics of individual isoflavones (daidzein, genistein, and glycitein) or their microbial metabolites (dihydrodaidzein, dihydrogenistein, equol, and *O*-desmethylangolensin).^59^ This could indicate abundant endogenous β-glucosidase along the gastrointestinal tract, sufficient to hydrolyze isoflavone glucosides. In a previous study with healthy women, even a higher bioavailability was observed when genistein and daidzein were administered as β-glycosides than their corresponding aglycones.^60^ It was also hypothesized that the glycosidic moiety could act as a protecting group to prevent biodegradation of the isoflavone structure. In this case, a delay in reaching the maximum concentration after the ingestion of isoflavone glucosides was observed, suggesting that the limiting factor in absorption was the initial hydrolysis of the glucoside. Higher bioavailability of glycosidic conjugates was also observed for quercetin, in this case, as a result of their higher solubility in water compared to quercetin aglycone.^61^ The bioavailability of quercetin glucosides can even be enhanced by enzymatic α-oligoglucosylation of their sugar moiety. Enzymatically modified isoquercitrin (quercetin-3-*O*-β-glucoside) (EMIQ) is a water-soluble glucoside of quercetin produced from rutin (quercetin-3-rutinoside) via enzymatic hydrolysis, which removes the rhamnosyl group, followed by treatment of the product with glycosyltransferase in the presence of dextrin to add glucose residues (1–7 of additional linear glucose moieties). A study with rats administered with quercetin aglycone and different quercetin glycosides showed that EMIQ exhibited the highest bioavailability among the glycosides examined, with a shorter *T*_max_ and higher *C*_max_ and AUC than any other form.^62^ The same results were observed in humans, where the plasma level of quercetin metabolites was instantly increased by oral intake of EMIQ. Its absorption efficiency (with higher *C*_max_ and AUC) was significantly higher than that of isoquercitrin and rutin.^63^ These data indicated that enzymatic α-oligoglucosylation of the sugar moiety is effective for enhancing the bioavailability of quercetin glucosides. The effectiveness of α-oligoglucosylation on the bioavailability of other flavonoids, such as hesperidin (hesperetin-7-*O*-rutinoside), had been previously demonstrated.^64^ Glucosyl hesperidin (G-hesperidin) was absorbed more rapidly and efficiently (higher *C*_max_ and AUC) than hesperidin because of its high water solubility.

In the case of phenolic rhamnosides (commonly found in the family of flavonoids), they are poorly absorbed. No endogenous hydrolysis at the small intestine level is produced, and they have to be hydrolyzed by gut microbiota before absorption.^65^ Two studies have shown that the removal of the rhamnose group to yield the corresponding flavonoid glucoside improves the bioavailability of aglycone. The increase in the bioavailability of hesperidin (hesperetin-7-*O*-rutinoside) after enzymatic treatment with rhamnosidase was demonstrated in a randomized double-blind clinical trial with subjects consuming orange juice or orange juice treated with hesperidinase (to yield hesperetin-7-glucoside).^66^ The peak plasma concentration (*C*_max_) of hesperetin was 4-fold higher and the AUC for total plasma hesperetin was 2-fold higher in subjects consuming enzymatically treated orange juice compared to standard orange juice. Besides, the absorption of hesperetin was much faster after enzymatic treatment (*T*_max_ of 0.6 h) compared to regular orange juice (*T*_max_ of 7 h), indicating a change in the absorption site from the colon to the small intestine. Similar results were observed with narirutin (naringenin-7-*O*-rutinoside). α-Rhamnosidase-treated orange juice showed higher AUC and *C*_max_ values in plasma (5.4- and 4-fold higher, respectively) and higher excretion in urine (6.7-fold) compared to untreated orange juice.^67^

In whole-grain cereal products, phenolic compounds are mainly in the bran fraction and covalently bound to cell wall polysaccharides. They show very low bioavailability limited by their bioaccessibility. The bran matrix hampers the access of the enzymes that release the phenolic compounds in the human gastrointestinal tract, reducing their absorption at the intestinal level. Phenolic compounds bound to the food matrix that are not absorbed reach the gut where they are metabolized by gut microbiota. Several strategies have been reported to increase the bioaccessibility of phenolic compounds, mainly ferulic acid, in cereal grains.^7,68,69^ Only two human intervention studies have evaluated the bioavailability of these phenolic compounds. The effect of enzymatic bioprocessing (consisted of a yeast fermentation combined with enzymatic treatment with cell-wall-degrading enzymes, mainly xylanase, β-glucanase, and feruloyl esterase) on the bioavailability of whole-meal bread phenolic compounds, previously evaluated *in vitro*,^70^ was also examined in a human study.^71^ The consumption of the bioprocessed bread led to an increase of different phenolic compounds, ferulic, vanillic, sinapic, and 3,4-dimethoxybenzoic acids, in plasma and urine samples compared to the control. In another study, the consumption of white wheat breads fortified with rye bran bioprocessed with enzymes (feruloyl esterase) and yeast increased the urinary excretion of ferulic acid (4-fold) compared to native bran. The increase in the absorption of ferulic acid from the small intestine is due to conversion of bound ferulic acid into free ferulic acid.^72^ No difference in microbial metabolites, benzoic, phenyl propanoic, and phenyl acetic acids, was observed between the breads, in agreement with the results found *in vitro*.^73^ More recently, Turner et al.^74^ demonstrated that enzymatic processing of high-fiber bread with Ultraflo L, a commercial β-glucanase that also possesses xylanase and feruloyl esterase activities, increased the bioavailability of ferulic acid with the higher plasma concentration at 2 h after consumption and led to improvements in human vascular function.

Other phenolic compounds that are poorly absorbed are those found in esterified form, such as caffeic acid, which occurs in plants mainly esterified as chlorogenic acid. Rivelli et al. used chlorogenate esterase to hydrolyze the phenolic content of an hydroethanolic extract of *Ilex paraguariensis*, rich in caffeoylquinic acid.^75^ Hydrolysis of the extract led to the conversion of all 5-caffeoylquinic acid into caffeic acid. Rats that ingested the enzymatically treated extract showed a much higher plasma concentration of caffeic acid than rats treated with the non-hydrolyzed extract. Besides, caffeic acid was found in the liver of the animals that received multiple doses of the hydrolyzed extract. Similar results were obtained in a randomized, double-blind, crossover study, in which healthy volunteers consumed three coffees with different degrees of roasting and an unroasted coffee enzymatically hydrolyzed with a purified esterase of the probiotic *Lactobacillus johnsonii* that releases caffeic acid from chlorogenic acid. After enzymatic hydrolysis, a larger quantity of phenolic acids was released from the coffee matrix. An increased absorption of phenolic acids in the small intestine was observed with the hydrolyzed unroasted coffee. These were most rapidly (*T*_max_ of 1 versus 9–11 h) and better absorbed (AUC 3-fold higher) compared to the unroasted coffees.^76^

### 3.2 Treatments with Microorganisms

These include microbial fermentations of food, co-administration of PPs with specific probiotics (synbiotics), and co-administration of PPs with specific gut bacteria to produce more bioavailable bioactive metabolites (postbiotic metabolites).

#### 3.2.1 Food-Based Fermentation

Fermentations using microorganisms capable of breaking down the complex phenolic compounds have been studied as a biotechnological option to enhance PP bioavailability.^77^ Fermentation can be spontaneous, with microorganisms present naturally, or can be forced using starters added purposely, which is more recommended to ensure a better control of the final product. Lactic acid bacteria have been used for a long time as fermentation starters to manufacture fermented foods.^78^ Many of them have demonstrated their ability to deglycosylate, de-esterify, decarboxylate, and demethylate dietary phenolic compounds.^79^ In this way, PPs can be biotransformed into compounds with enhanced bioavailability and bioactivity. Several studies have focused on changes of the phenolic profiles of foods over fermentation with different microorganisms, demonstrating in many cases that microbial fermentation increases the proportion of aglycones.^80−82^ The ability of fermentation to increase antioxidant capacity^83−85^ and other biological activities^86,87^ of phenolic-rich food was also observed. However, few *in vivo* studies have provided relevant information regarding the bioavailability and metabolism of PPs following fermentation (Table 4). Most of them explored the effect of fermentation on bioavailability and metabolism of isoflavones from soy products. Several studies have demonstrated that isoflavone aglycones present in fermented food showed an improved bioavailability and bioactivity compared to the original glucosides because they are more lipid-soluble and, thus, easily able to go through the intestinal barrier.^88^ Enhanced isoflavone bioavailability was also observed in different studies with ovariectomized mice after consumption of fermented soybean products.^78,89^ In healthy adults, Hutchins et al. reported that the fermentation of cooked soybeans by *Rhizopus oligosporus* (tempeh) enhanced the bioavailability of daidzein and genistein over a 9 day feeding period compared to the ingestion of non-fermented cooked soybean.^90^ In another study with humans, the changes in soybean isoflavones caused by fermentation, increasing simple and acylated glucoside levels, resulted in faster absorption and higher bioavailability of some metabolites in plasma after consumption of fermented soybean.^91^ Although the 24 h urinary excretion of total isoflavone metabolites did not significantly differ between fermented and non-fermented samples, changes in the isoflavone conjugate profile were observed, finding genistein 7-*O*-sulfate as a discriminant metabolite for the fermented soybean.

**Table 4:** Human Studies with Fermented Foods To Improve the Bioavailability of PPs^a^

fermented food	polyphenols	fermentation starter	volunteers	results (fermented versus control)	reference
soybeans (tempeh)	isoflavones and lignans	*Rhizopus oligosporus*	healthy men (*n* = 17)	↑ urinary recoveries of daidzein (1.70-fold) and genistein (1.46-fold)	(90)
soybean	isoflavones	*Bacillus subtilis* KACC18604	healthy (*n* = 10)	↑ AUC in plasma of dai-7G-4′S (1.20-fold) and gen-4′,7-diG (1.33-fold)	(91)
				no changes in 24 h urinary excretion of total isoflavones but genistein 7-*O*-sulfate discriminant metabolite for the fermented soybean	
soymilk	isoflavones	*Bifidobacterium breve* and *Lactobacillus mali*	healthy (*n* = 12)	↑ AUC in plasma of daidzein (1.5-fold) and genistein (2.3-fold), and concentrations were reached more quickly (*T*_max_ of 1 versus 6 h)	(92)
				↑ urinary excretion of isoflavones (1.2-fold daidzein and 1.4-fold genistein)	
soymilk	isoflavones	*Lactobacillus casei*	healthy premenopausal (*n* = 7)	↑ AUC in plasma of daidzein (1.3-fold) and genistein (1.4-fold)	(93)
soymilk	isoflavones	*Bifidobacterium animalis* Bb-12	healthy postmenopausal (*n* = 16)	similar levels of total isoflavones in urine	(95)
				no evidence of improved bioavailability	
cabbage	anthocyanins		healthy (*n* = 13)	↓ AUC in plasma (1.3-fold) and urine (1.4-fold) of anthocyanins	(97)
				↓ antioxidant capacity in plasma	
red wine	anthocyanins		healthy (*n* = 9)	↓ urinary excretion (1.3-fold) and AUC in plasma (1.6-fold) of individual and total anthocyanins	(98)
orange juice	flavanones and phenolic acids	Saccharomycetaceae *Pichia kluyveri*	healthy (*n* = 9)	fermentation did not influence the pharmacokinetic parameters and urinary excretion of (poly)phenol metabolites but faster absorption	(99)

a dai-7G-4′S, daidzein 7-*O*-glucuronide-4′-*O*-sulfate; gen-4′,7-diG, genistein 4′,7-di-*O*-glucuronide.

In studies with fermented soymilk, an increase in the serum concentration and urinary excretion of isoflavones was observed in healthy volunteers compared to the consumption of non-fermented products.^92,93^ These results demonstrated that the isoflavone aglycones of soymilk were absorbed faster and in greater amounts than their glucosides. In mice consuming fermented soymilk, an increase in the urinary excretion of the isoflavone metabolites, *O*-desmethylangolensin (*O*-DMA) and equol, was observed.^94^ In contrast, another study showed no strong evidence to suggest that fermenting soymilk with bifidobacteria improved the bioavailability of isoflavone in postmenopausal women over 14 days of daily soymilk ingestion.^95^ Levels of total isoflavone excreted in urine were similar for women consuming either fermented or non-fermented soy beverages.

The bioavailability of other families of PPs present in fermented products have also been studied. The fermentative process in a turmeric beverage administered to rats resulted in an increase in antioxidant activity and total PP concentration in plasma.^96^ In contrast, fermentation of red cabbage showed lower anthocyanin bioavailability and plasma antioxidant capacity compared to fresh cabbage consumption in a randomized crossover human study.^97^ In this case, the fermentation process reduced red cabbage anthocyanin bioavailability and human plasma antioxidant capacity. A reduction in anthocyanin bioavailability was also observed after consumption of equal amounts of red wine compared to red grape juice.^98^ Higher urinary excretion of total anthocyanins in the glucoside form was observed in the case of juice (0.23%) than in wine (0.18%), and the relative bioavailability of five individual anthocyanins (glucosides of cyanidin, delphinidin, malvidin, peonidin, and petunidin) tended to be higher in the juice according to plasma pharmacokinetic parameters. The authors suggested that ethanol produced by fermentation could affect the accessibility of these red grape PPs. This was not confirmed by Bub et al., who observed a similar bioavailability of malvidin-3-glucoside after consumption of regular red wine and that without alcohol.^200^ In another study, the effect of a controlled alcoholic fermentation in the bioavailability of orange juice PPs was examined after accurate administration to nine volunteers. The fermentation did not influence the pharmacokinetic parameters and urinary excretion of the PP metabolites, but PPs in the fermented juice were absorbed faster than after orange juice intake.^99^ The lack of differences on the pharmacokinetic parameters, despite the effects on the absorption profile, could be related to the high variability observed.

#### 3.2.2 Symbiotic

Another approach is the co-administration of PPs with selected probiotic strains. The probiotic in the co-administration can increase bioavailability by two different ways: through a direct hydrolysis of PPs increasing their bioavailability or through the modification of the gut microbiota composition in a manner that significantly affects PP bioavailability or metabolism.

The effect of co-administration with probiotics has been observed in different preclinical studies. Anthocyanins from berries supplemented in a mice model with *Lactobacillus plantarum* HEAL19 showed a trend to decrease in the cecum and colon, suggesting a possible increase in metabolic activity of gut microbiota in the presence of the probiotic, although no significant differences in the concentration of phenolic metabolites were observed.^100^ Significantly increased concentrations of several microbial metabolites, *p*-coumaric, *m*-coumaric, and *p*-hydroxybenzoic acids, were observed in mice plasma after co-supplementation of phenolics from a cranberry extract with spores of *Bacillus subtilis* CU1.^101^ These changes were associated with significant variations in their gut microbiota (increase of *Barnesiella* and decrease of *Oscillibacter*). Authors hypothesized that the increase in the microbial metabolites is more likely due to gut microbiota reshaping rather than the direct action of this probiotic on the phenolic compounds of the cranberry extract. A higher concentration of metabolites (hydroxytyrosol sulfate, coumaric acid sulfate, and ferulic acid sulfate) was also observed in the urine from healthy mice after co-administration of *Lactobacillus plantarum* 299v and a standardized extract of *Olea europaea* leaves. In this case, the increase was attributed to the improvement of *in vivo* conversion of oleuropein to hydroxytyrosol by this probiotic.^102^ In another study, pharmacokinetic analyses revealed that the co-administration of *Lactobacillus paracasei* 221 and kaempferol-3-sophoroside significantly enhanced the amount of deconjugated kaempferol in murine plasma samples at 3 h post-administration.^103^

In the case of human studies, few works have been found (Table 5), mainly with isoflavones, and in some cases, the results are not conclusive. The effect of probiotic co-administration (10^9^ CFU of *Lactobacillus acidophilus* and *Bifidobacterium longum*) on the bioavailability of soy isoflavones, consumed as soy protein, was studied for the first time in a 6 week crossover trial with 40 postmenopausal women.^104^ Plasma phytoestrogen concentrations (daidzein, genistein, equol, and ODMA) and the number of equol producers were unaffected by this particular probiotic supplement, with the exception of two volunteers who changed their equol producer status. Besides, within the group of equol producers (*n* = 8), 67% showed increased equol excretion when consuming the probiotics, although there were not consistent changes in daidzein or ODMA to explain how isoflavone metabolism could be affected. Larger populations are needed to corroborate these results. No effect in urinary equol excretion was observed in another study with premenopausal women (*n* = 34) consuming soy protein co-administered with probiotic capsules containing 10^9^ CFU of *L. acidophilus* and *B. longum* for 2 months.^105^ These probiotic bacteria may not be the right bacteria to successfully alter phytoestrogen metabolism. In fact, other bacterial genera have been described to be responsible for equol production.^106,107^

**Table 5:** Effect of Co-administration with Probiotics in the Human Bioavailability of Polyphenols

matrix (polyphenols)	probiotic strain	volunteers	results (probiotic versus control)	reference
soy protein (isoflavones)	3 caps/day of *Lactobacillus acidophilus* and *Bifidobacterium longum* at 10^9^ CFU (6 weeks)	healthy postmenopausal (*n* = 40) (20 breast cancer survivors and 20 without breast cancer history)	no significant difference in plasma phytoestrogen concentration (daidzein, genistein, equol, and ODMA); number of equol producer unaffected; 67% of the equol producers (*n* = 5) showed an increase in equol urinary excretion	(104)
soy protein (isoflavones)	3 caps/day of *Lactobacillus acidophilus* and *Bifidobacterium longum* at 10^9^ CFU (2 months)	healthy premenopausal (*n* = 34)	no significant difference in urinary equol excretion	(105)
high soy diet (isoflavones)	1 yogurt/day containing 10^8^ CFU of *Lactobacillus acidophilus*, *Bifidobacterium bifidus*, and *Lactobacillus* GG (5 weeks)	at least 45 years old and mildly hypercholesterolemic men and postmenopausal (*n* = 31)	no significant difference in plasma and urine concentrations of genistein, daidzein, and equol	(108)
soy formulation (isoflavones)	4 caps/day *Lactobacillus* GG at 10^12^ CFU (3 weeks)	healthy premenopausal (*n* = 32)	no significant decrease in genistein and daidzein urinary excretion (no equol measurement)	(109)
orange juice (flavanones)	*Bifidobacterium longum* R0175 at 10^9^ CFU acute or chronic (5 weeks)	healthy volunteers: acute study (*n* = 27) and chronic study (*n* = 16)	acute: no significant effect in urinary excretion	(110)
			chronic: increase of urinary excretion of flavanone metabolites (1.3-fold) and colonic metabolites (2-fold)	

In another study, the concurrent consumption of a high soy diet with a probiotic (yogurt containing 10^8^ CFU of *L. acidophilus*, *Bifidobacterium bifidus*, and *Lactobacillus* GG) for 5 weeks did not significantly alter plasma and urinary daidzein, genistein, or equol concentration or the equol-producing ability of the subjects in this study.^108^ There were trends, although not significant, for subjects who produced equol to have higher plasma concentrations of daidzein, genistein, and equol after the probiotic treatment. In a study with 32 premenopausal women, the co-administration of a high concentration of a probiotic (10^12^ CFU of *Lactobacillus* GG) with a soy formulation for 1 month reduced the urinary excretion of total and individual isoflavones (daidzein and genistein) by 40%.^109^ A possible alteration of the isoflavone metabolism was suggested. However, the lack of information about isoflavone blood levels and urinary equol and ODMA concentrations made it impossible to conclude the exact effect of the probiotic. A potential effect on isoflavone deconjugation or a suppression of their degradation remains yet to be demonstrated.

Pereira Caro et al. examined the acute (5 days; *n* = 27) and chronic effects (33 days; *n* = 16) of orally administered *B. longum* R0175, a probiotic known for its rhamnosidase activity, on the bioavailability of orange juice flavanones.^110^ Results were similar when orange juice was consumed with and without an acute probiotic intake: the urinary excretion of hesperetin and naringenin metabolites, such as hesperetin-*O*-glucuronide, naringenin-*O*-glucuronide, and hesperetin-3′-*O*-sulfate, corresponded to 22% of the flavanone intake, and the excretion of colon-derived phenolic and aromatic acids was 21%. However, after chronic administration of the probiotic, PP recovery in urine increased to 27% for flavanone metabolites and 43% for colonic metabolites, leading to a total excretion of 70% of the ingested orange juice PPs. This study highlighted the positive effect of chronic but not acute intake of a probiotic on the bioavailability of orange juice flavanones.

#### 3.2.3 Postbiotic Metabolites

In many cases, PP bioavailability is mediated by gut microbiota, and therefore, the metabolites produced can be considered “postbiotic metabolites”. Good examples for this are the citrus flavanones (flavanone rutinosides), the oligomeric proanthocyanidins, the hydrolyzable tannins (gallotannins and ellagitannins), ellagic acid, lignans, and isoflavones. The bioavailability of these PP-derived postbiotic metabolites is generally much higher than that of the PPs occurring in foods.^111^ For these reasons, the methods that facilitate the production of postbiotic metabolites also favor the bioavailability and finally the biological effects of PPs. Thus, methods that enhance the production of postbiotic metabolites that include many of those reviewed in the previous sections can be an excellent strategy to improve bioavailability and health effects of dietary PPs.

Postbiotic metabolites also include short-chain fatty acids (SCFAs) that are produced by probiotic bacteria and other gut microbes from the complex carbohydrates present in dietary fiber. SCFAs have been shown to enhance the absorption and bioavailability of dietary PPs and their gut microbiota metabolites.^112^ It is clear now that the biological effects of PPs in humans are often carried out through the interaction with gut microbiota. This is a two-way interaction^10^ in which gut microbiota is modulated by the ingested PPs producing a “prebiotic-like” effect^113,114^ and gut microbiota transform PPs into bioavailable and bioactive metabolites that could be included in the frame of “postbiotic metabolites”.^115^ If the bioavailability of PP-derived postbiotic metabolites can also be considered as part of PP bioavailability, then those factors that improve the production of PP postbiotic meabolites can be considered as enhancers of PP bioavailability *sensu lato*. In addition, it has been demonstrated that the PP gut microbiota metabolites are much better absorbed than the original PPs and show relevant systemic biological effects.^111^ Therefore, the way that PPs are present in the food product can heavily impact their interaction with gut microbiota and can affect their prebiotic-like effects and the production of postbiotic metabolites that are generally much more bioavailable than the original PPs.^116^ Some examples could be considered to illustrate the relevance of methodologies than can increase the production of postbiotic metabolites. These could include urolithins, hesperetin, lignans, and valerolactones.

In conclusion, the impact of technological and biotechnological processes on the bioavailability of different families of phenolic compounds in humans has been minimally studied. Food processing, particularly thermal processing, plays a significant role in the bioavailability of PPs and, in some cases, could be used as a strategy to enhance PP bioavailability. However, the variability of studies was observed depending upon the type of matrix, the processing conditions applied, and to a lesser extent the analytical method used for the analysis of polyphenols. Several PP formulations based on nanotechnology have demonstrated an improved bioavailability in different *in vitro* and *in vivo* models with animals. However, only a few formulations, mainly focused on curcumin, showed higher bioavailability in humans, and many questions and challenges persist considering oral administration. With regard to biotechnological processes, enzymatic hydrolysis has demonstrated to be a good approach to enhance the bioavailability of glucoside and rhamnoside derivatives, phenolic acids entrapped in the cereal matrix, or other phenolics found in esterified forms, such as caffeic acid. Fermentation has also been demonstrated to be a good strategy to enhance the bioavailability of PPs, mainly isoflavones from soy products. With regard to co-administration with probiotics, although results in *in vitro* gastrointestinal simulators provide evidence that the probiotic strain may improve the metabolism of dietary PPs, the few *in vivo* studies showed inconsistent results. In general, more human studies addressing more families of phenolic compounds should be considered to obtain conclusive results.

## Supporting Information Available

Rat pharmacokinetic studies to evaluate polyphenol bioavailability under different formulations (Supplementary Table 1) (PDF)
