# Supplementary material for: Technological and Biotechnological Processes To Enhance the Bioavailability of Dietary (Poly)phenols in Humans
Source: J Agric Food Chem. 2022 Feb 14;70(7):2092–107. doi: 10.1021/acs.jafc.1c07198 (PMC8880379; doi:10.1021/acs.jafc.1c07198)
Supplement: Supplementary file 1 — jf1c07198_si_001.pdf [file jf1c07198_si_001.pdf]

Supplementary Table 1. Rat pharmacokinetic studies to evaluate polyphenol bioavailability under different formulations

Polyphenol	Type of study	Oral delivery system	Material	Results	References
**Curcumin**					
	Wistar rats (n=18, 3 groups)	Mixed micelles by a solvent evaporation method	Pluronic F-127 and Gelucire®44/14	↑ AUC (55.7-fold); Cmax (3 -fold); tmax (2-fold)	1
	SD rats (n=30, 2 groups)	Nanocapsules	Self –aggregates of Octenylsuccinate oat β-glucan	↑ AUC (1.4-fold); Cmax (1.4 -fold); =tmax	2
	SD rats (n=12, 2 groups)	Nanoparticles by the pH-driven method (Micelles)	Sophorolipid	↑ AUC (4.6-fold); Cmax (5.8 -fold); =tmax	3
	SD rats (n=12, 2 groups)	Nanoparticles	Bowman-Birk inhibitor (BBI)	↑ AUC (11-fold); Cmax (11.4 -fold); tmax (2-fold) (vs sodium caseinate nanoparticles)	4
	SD rats (n=10, 2 groups)	Nanoparticles by solid-in-oil-in-water (s/o/w) solvent evaporation method	PLGA	↑ AUC (5.6 fold); Cmax (4.4 -fold); tmax (1.2-fold)	5
	Wistar rats (n=15, 3 groups	Nanoparticles by single-emulsion solvent-evaporation technique	PLGA and PLGA-PEG	PLGA: ↑ AUC (15.6-fold); Cmax (2.9 -fold); tmax (4-fold)<br>PLGA-PEG: ↑ AUC (55.5-fold); Cmax (7.3 -fold); tmax (6-fold)	6
	SD rats (n=15, 3 groups)	Nanoparticles by emulsion-diffusion-evaporation method	PLGA	PLGA-nanoparticles: ↑ AUC (10.3-fold); Cmax (2.9 -fold); tmax (4-fold)<br>Cur-piperine susp: ↑ AUC (2.8-fold); Cmax (1.3 -fold); tmax (1.5-fold)	7
	Rats	Inclusion complex by water-ethanol cosolvent incubation-lyophilization	Hydroxypropyl-β-cyclodextrin (HP-β-CD)	↑ AUC (2.77-fold)	8
	ICR mice (n=60, 2 groups)	Emulsions	Casein and soy soluble polysaccharide complex	↑ AUC (3.1-fold); Cmax (1.6 -fold); ↓tmax (8-fold)	9

	Wistar rats (n=45, 15 groups)	Coated nanoemulsions (effect of piperine co-delivery)	Soy-bean oil , Lipoid E80 lecithin (oil in water nanoemulsion); Thiol modified chitosan (coating)	↑ AUC (33-fold cur; 85.5-fold cur-glucu;6.1-fold cur-sulf) Piperine: ↑ AUC (63.6-fold cur; 42.4-fold cur-glucu; 4.3-fold cur-sulf)	10
	Kunming mice (n=10, 2 groups)	SMEDDS	Ethyl oleate (oil), OP:Cremorphor EL 1:1 (surfactant) and PEG 400 (co-surfactant)	↑ Absorption percentage (3.86-fold)	11
	CD-1 mice (n=6, 2 groups)	Organogel-based nanoemulsions	Span 20-saturated MCT and monostearin(organogel), Tween 20 (emulsifiers)	↑ AUC (9.8-fold Cur;8.5-fold D-Cur); Cmax (18.7 –fold Cur; 14.8-fold D-Cur) = tmax	12
	Wistar rats (n=12, 2 groups)	Phospholipid complex	Hydrogenated soy phosphatidylcholine	↑ AUC (5.2-fold); Cmax (2.4 -fold); tmax (2-fold)	13
	SD rats	Phospholipid complex	Soya phospholipid	↑ AUC (3.4-fold); Cmax (2.3 -fold); tmax (1.4-fold)	14
	Wistar rats (n=30, 2 groups)	Phospholipid-complex (MERIVA)	Soybean lecithin enriches in phosphatidylcholine	↑ AUC (5.6-fold Cur; 23.8-fold Cur-glucu; 1.6-fold-Cur-sulf); Cmax (5.1 –fold Cur; 19.6-fold Cur-glucu; 3-fold Cur-sulf); ↓tmax (2-fold Cur)	15
	SD rats (n=21, 3 groups)	LIposome	Soybean Lecithins	↑ AUC (5fold); Cmax (5 -fold); ↓tmax (40-fold)	16
	Rats	Uncoated and coated liposomes by a thin –film dispersion method	Soybean phosphotidylcholine, cholestrol, and TPGS (liposomes), chitosan and N-trimethyl chitosan chloride (coating)	↑ AUC (1.07-fold uncoated;1.7-fold coated liposomes)<br>↑ Cmax (0.90-fold uncoated;1.3-fold coated liposomes)<br>↑ t1/2 (2.5-fold uncoated;3.1-fold coated liposomes)	17
	Wistar rats (n=30, 5	SLNs by microemulsification technique	Polysorbate 80 and soy lecithin	↑ AUC (39-fold); Cmax (49 -fold); tmax (2-fold)	18

	groups)				
	Balb/c mice (n=12, 4 groups)	Uncoated and Coated SLNs	Palmitic acid, cholesterol and TPGs (SLNs); chitosan and N-trimethyl chitosan (coating)	↑ AUC (19.4-fold SLNs;22.4-fold CH-SLNs; 41.2-fold TMC-SLNs)<br>↑ Cmax (2.4-fold SLNs;2.8-fold CH-SLNs; 5-fold TMC-SLNs)<br>↑ tmax (4-fold all formulations)<br>↑ MRT (2.8-fold SLNs and CH-SLNs; 3.2-fold TMC-SLNs)	19
	SD rats (n=16, 4 groups)	Nanosuspension	TPGS, Brij78, and Pluronic-F68 (stabilizers)	↑ AUC (3.7-fold CUR/Brij78;3.2-fold CUR/TPGS; 1.3-fold CUR/F68)<br>↑ Cmax (21.6fold CUR/Brij78;12.5-fold CUR/TPGS; 2.5-fold CUR/F68)<br>↓ tmax (2-fold in all formulations)	20
	SD rats (n=12, 2 groups)	Nanosuspension by the precipitation-high speed homogenization method	Poloxamer-188 (stabilizer)	↑ AUC (4.2-fold); Cmax (7.6 -fold) ↓tmax (2-fold)	21
	Mice	Nanosuspension	TPGS (stabilizer)	↑ AUC (6.8-fold);	22
	SD rats (n=42, 7 groups)	6 formulations: micronized suspension , nanosuspension, amorphous solid dispersion, HP-β-CD inclusion complex, combination with piperine, spray-dried CRM-milk composite	Na-CMC, Poloxamer, PVP, HP-β-CD, piperine and milk, respectively	↑ AUC (2.51-fold nanosuspension; 5.67-fold HP-β-CD complex, 4.45-fold amorphous solid dispersion)<br>=AUC with piperine and micronized suspension<br>↓AUC milk composite<br>↑tmax with piperine and HP-β-CD complex<br>↓ tmax nanosuspension	23
**Flavonoids**<br>**Flavanones**					
Naringenin	Wistar rats (n=12, 2 groups)	SNEDDS	Triacetin (oily phase), Tween 80 (surfactant) and Transcutol HP (co-surfactant)	↑ AUC (2.82-fold); Cmax (4.93-fold)<br>↓ tmax (2-fold)	24
Naringenin	SD rats (n=12,	Liposomes by thin-film	Phospholipid, cholesterol,	Conjugated: ↑ AUC (13.44-fold); Cmax (10-	25

	2 groups)	hydratation method	sodium cholate, and isopropyl myristate	fold); MRT (1.1-fold); tmax (3-fold)<br>Free: ↑ AUC (6.9-fold); Cmax (6.5-fold); MRT (1.2-fold); =tmax<br>↑ concentration in different tissues, especially liver	
**Flavonas**					
Apigenin	Wistar rats (n=12, 3 groups)	Water/oil/water emulsions (W/O/W)	Soybean oil and Tween 80	↑ Cmax (9-fold);	26
Baicalin	SD rats (n=20, 4 groups)	Liposomes by effervescent dispersion technique	Tween® 80, Phopholipon® 90 H, citric acid	↑ AUC (3-fold); Cmax (2.8-fold); MRT (0.9-fold); tmax (2-fold)<br>↑ concentration in the liver (5.6-fold), kidney (2.3-fold) and lung (1.3-fold)	27
Baicalin	Wistar rats (n=12, 2 groups)	SLNs by coacervation	Stearic acid alkaline salt	↑ AUC (2.6-fold); Cmax (1.6-fold); MRT (1.9-fold<br>= tmax	28
Baicalin	SD rats (n=30, 3 groups)	Nanoemulsions	Soy-lecithin, tween-80, polyethylene glycol 400 and isopropyl myristate	↑ AUC (7-fold); Cmax (2.7-fold); MRT (3.6-fold); tmax (6-fold)	29
Baicalin	Rats	Nanoemulsion		↑ AUC (14.6-fold);	30
Baicalin	Wistar rats (n=6, 2 groups)	Micelles	Pluronic P123 copolymer and sodium taruocholate	↑ AUC (1.5-fold); Cmax (1.7-fold); tmax (1.7-fold): =MRT	31
Baicalin	SD rats (n=12, 2 groups)	Inclusion complex	β-cyclodextrin	↑ AUC (2.5-fold); Cmax (2.2-fold); ↓tmax (1.2-fold)	32
Baicalin	Beagle dogs (n=6) cross over	Solid dispersion	Polyvinylpyrrolidone	↑ AUC (3.2-fold); Cmax (4.6-fold); ↓tmax (15-fold); MRT(1.2-fold)	33
Baicalin	SD rats (n=12, 2 groups)	Combination of phospholipid complex (PC) and SMEDDS	Ethyl oleate, Tween-80 and glycerol	↑ AUC (2.3-fold); Cmax (2-fold); tmax (1.3-fold)	34
**Flavonols**					

Fisetin	C57BL6 mice (n=44, 2 groups)	Encapsulation as inclusion complex in polymeric nanoparticles	HPβCD (inclusion complex) and PLGA (polymer)	↑ AUC (15.6-fold); Cmax (8.8-fold); MRT (1.7-fold); tmax (1.3-fold)	35
Quercetin	Wistar rats (n=10, 2 groups)	SLNs by emulsification and low- temperature solidification	Glyceryl monostearate (GMS) and soya lecithin	↑ AUC (5.7-fold); Cmax (2-fold); MRT (2.2-fold); tmax (1.6-fold)	36
Quercetin	Wistar rats (n=10, 2 groups)	Nanosuspension by nanoprecipitation and high pressure homogenization	Lecithin as stabilizers	↑ AUC (15.6-fold); Cmax (2.1-fold); MRT (10.2-fold); tmax (2.7-fold)	37
Persimmon leaf extracts (quercetin + kampferol)	Beagle dogs (n=5) Cross over study	SNEDDS	Cremophor EL, Transcutol P, Labrafil M 1944 CS (56:34:10. w/w)	Quercetin: ↑ AUC (1.5-fold); Cmax (1.6-fold); = MRT; ↓tmax (1.25-fold) Kaempferol: ↑ AUC (1.6-fold); Cmax (1.3-fold); =MRT ; ↓tmax (1.25-fold)	38
Quercetin	Rats	SNEDDS	Castor oil, Tween® 80, Cremophor® RH 40, and PEG 400 (20:16:34:30, w/w)	↑ AUC (2-fold); Cmax (3-fold);	39
Quercetin	SD rats (n=12, 2 groups)	SEDDS	Capmul MCM (oily phase), Tween 20 (surfactant) and ethanol (cosurfactant)	↑ AUC (5-fold); Cmax (7.8-fold) = tmax	40
Quercetin coencapsulated with tamoxifen	SD rats (n=20, 4 groups)	Nanoparticles	PLGA	↑ AUC (2.9-fold); Cmax (2.6-fold); ↓tmax (1.33-fold)	41
Quercetin	Beagle dogs (N=6, two groups)	Polymeric micelles by a modified film dispersion method	Soluplus	↑ AUC (2.86-fold); Cmax (1.4-fold); MRT (3.7-fold); tmax (1.3-fold)	42
Quercetin	C57BL6 mice (n=44, 2 groups)	Cationic nanostructured lipid carriers by emulsifying (high T) and solidifying (low T)	Glycerol monostearate (GMS), medium chain triglycerides (MCT) and soy lecithin	↑ AUC in lung (1.6-fold), liver (1.5-fold) and kidney (1.7-fold)	43
Quercetin	Wistar rats	Nanoparticles alone or in combination with HP-β-CD	Zein	**NP:** ↑ AUC (9-fold); Cmax (2.6-fold); MRT (2.5-fold); tmax (8-fold)	44

				**HPCD-NP:** ↑ AUC (14-fold); Cmax (2.4-fold); MRT (5.2-fold); tmax (9-fold)	
Quercetin	Wistar rats (n=30, 5 groups)	Nanoparticles by coacervation alone or in combination with HP-β-CD	Casein	**NP:** ↑ AUC (2.9-fold); Cmax (1.6-fold); MRT (1.6-fold); tmax (6.2-fold)<br>**HPCD-NP:** ↑ AUC (9-fold); Cmax (1.4-fold); MRT (5.8-fold); tmax (10.5-fold)	45
**Isoflavones**					
Isolflavone extract	SD rats (n=6, 2 groups)	Cyclodextrin complex	β-cyclodextrin	↑ AUC daidzein (1.3-fold), genistein (1.8-fold) , glycitein (1.7-fold)	46
Daidzein	SD rats (n=8, 2 groups)	SMEDDS	Ethyl oleate, Cremophor RH 40 and polyethylene glycol 400 (PEG400) (30%) (10:60:30, w/w)	↑ AUC (2.5-fold); Cmax (2.6-fold); ↓MRT (1.32-fold); tmax (1.4-fold)	47
Daidzein	SD rats (n=18, 3 groups)	Phospholipid complex and those encapsulated into SLNPs by a film-homogenization technique	Soybean phospholipids, glycerol monostearate and sodium oleate	**PC:** ↑ AUC (3.6-fold); Cmax (10-fold); ↓tmax (7.14-fold)<br>**SLNPs:** ↑ AUC (6.8-fold); Cmax (21-fold); ↓tmax (12.5-fold)	48
Genistein	SD rats (n=10, 2 groups)	Complexation	High-amylose corn starch	↑ AUC (1.6 fold); Cmax (1.5-fold);<br>↑urine concentration (3.4-fold)<br>↓ feces concentration (2.8-fold)	49
Genistein	Rats	Mixed micelles by organic solvent evaporation	Soluplus® and Vitamin E d-α-tocopheryl polyethylene glycol 1000 succinate (TPGS)	↑ AUC (2.4-fold);	50
Puerarin	SD rats (n=12, 2 groups)	SLNPs by solvent injection methods	Monostearin and soya lecithin (organic phase); 0.5% poloxamer 188 (aqueous phase)	↑ AUC (3.1-fold); Cmax (2 -fold); MRT (1.8-fold); ↓tmax (2.78-fold)<br>↑ AUC liver (4.7-fold), spleen (3.8-fold), brain (2.3-fold), heart (1.8-fold), kidney (1.2-fold), lung (1.4-fold)<br>↑urine concentration<br>↓feces concentration	51
**Flavan-3-ols**					

EGCG	SD rats (n=6, 2 groups)	Lipid-coated nanoparticles (nanolipidic particles) by co-solubilization methodology	Proprietary starting materials	↑ AUC (2.3-fold); Cmax (5.1 -fold); tmax (2-fold)	52
EGCG	Swiss Outbred mice (n=6, 2 groups)	Nanoparticles	Chitosan-tripolyphosphate	↑ AUC (1.5-fold); Cmax (1.1 -fold); = tmax	53
Catechin	Rats	Liposomes by thin film method	Soy phosphatidylcholine, cholesterol, and Tween 80	↑ AUC and tmax<br>↑ Brain concentration (2.9-fold in cerebral cortex and 2.7-fold in hippocampus)	54
Catechin	Wistar rats (n=12, 3 groups)	Liposomes and chitosomes (liposomes coated with chitosan)	Phosphatidyl choline, phosphatidyl serine and chitosan	Liposomes: ↑ AUC (1.5-fold); Cmax (1.3 -fold); tmax (1.5-fold)<br>Chitosomes: ↑ AUC (2.1-fold); Cmax (2.0 -fold); tmax (4-fold)	55
Catechin	Wistar rats	SDEDDS	Labrafac (oil), oleic acid (fatty acid) and Tween 80 (surfactant)	↑ AUC (2.1-fold);	56
Tea polyphenols extract	SD rats (n=12, 2 groups)	Nanoemulsions by high-pressure homogenization	Polysorbate 80 and corn oil	ECGC: ↑ AUC (1.3-fold); ↓ Cmax (1.6 -fold); = tmax<br>EGC: ↑ AUC (1.3-fold); Cmax (2.3 -fold); tmax (6-fold);<br>ECG: ↓ AUC (2-fold); Cmax (2.6 -fold); = tmax	57
**Resveratrol**					
Fish oil, tributyrin and resveratrol (radiolabeled)	SD rats (n=120, 6 groups)	Microencapsulation in oil-in –water emulsion	Sodium caseinate, high amylose maize starch and glucose	Relative distribution along GI were not markely altered<br>↑ levels of radioactivity in blood and liver	58
	Wistar rats (n=6, 2 groups)	Nanoparticles by nanoprecipitation	Eudragit RL polymer	↑ AUC (7.25-fold); Cmax (1.29-fold); MRT (8.82-fold); tmax (12-fold)<br>↑concentration at longer times in heart,	59

				lungs, kidneys, brain and especially in liver and spleen	
	Rats	Nanoparticles by nanoprecipitation	PLGA	↑ AUC (10.8-fold)<br>↑liver concentration (2.78-fold)	60
	Wistar rats (n=24, 4 groups)	Nanoparticles by desolvation method and spray-drying	Zein	↑ AUC (18.5-fold); Cmax (1.95-fold); MRT (13-fold); tmax (8.23-fold)	61
	Wistar rats (n=24, 4 groups)	Nanoparticles by coacervation and spray-drying	Casein	↑ AUC (10-fold); Cmax (1.45-fold); MRT (6.3-fold); tmax (3-fold).<br>↑ability to reach intestinal epithelium (biodistribution study)	62
	SD rats (n=6, 2 groups)	Nanoparticles prepared by emulsion cross-linking	Carboxymethyl chitosan	↑ AUC (3.6-fold); Cmax (1.2-fold); tmax (2.12-fold)	63
	Wistar rats (n=10, 2 groups)	SLNs coated with poloxamer 188 by solvent diffusion-solvent evaporation method	Stearic acid and Phospholipon®90G	↑ AUC (8-fold); Cmax (1.61-fold); MRT (3.8-fold); tmax (4-fold)	64
	Balb/c mice (n=9, 3 groups)	Surface-modified SLNPs prepared by emulsification and ultrasonication	Precirol ATO5, palmitic acid, Gelucire 50/13 (lipid phase) and N-trimethyl chitosan conjugated with palmitic acid (surface modifier)	TMC-g-PA: ↑ AUC (3.8-fold); Cmax (1.6-fold); MRT (1.6-fold); tmax (5.3-fold)<br>SLNPs: ↑ AUC (2.2-fold); MRT (1.5-fold); tmax (2.6-fold); =Cmax	65
	Wistar rats (n=6, 2 groups)	SNEDS	Lauroglycol FCC (lipid), Labrasol and Transcutol P (surfactant)	↑ AUC (4.31-fold); Cmax (2.34-fold)	66
	SD rats (n=8, 2 groups)	Carrier-free nanoparticles by ultra-nanoprecipitation method	SDS	↑ AUC (2.36-fold); Cmax (2.7-fold)	67
**Phenolic acids**					
Chlorogenic acid	Wistar rats (n=18, 2 groups)	Nanoparticles by ionic gelation method	Chitosan	↑ AUC (1.6-fold); MRT (2.3-fold); tmax (2.7-fold)<br>↓Cmax (1.62-fold)	68

Ferulic acid	Wistar rats (n=12, 2 groups)	Nanoparticles by ionic gelation method	Chitosan	↑ tmax in plasma (6-fold) and urine (4-fold)<br>↑plasma concentration between 4-6 h and still detected after 8 h	69
Syringic acid	SD rats (n=6, 2 groups)	Liposome by thin film dispersion method	Lecithin, cholesterol and vitamin E TPGS	↑ AUC (2.8-fold); MRT (2.10-fold); t1/2 (2.83-fold)<br>= Cmax, tmax	70
**Ellagic acid**	Wistar rats (n=12, 3 groups)	SNEDDS	Polyethylene glycol, polysorbate, caprylic/capric triacylglycerol (45:45:10)	↑ AUC (6.6-fold); Cmax (9.61-fold)<br>= tmax	71
	Wistar rats (n=12, 2 groups)	Phospholipid –complex	Hydrogenated soy phosphatidylcholine	↑ AUC (2.85-fold); Cmax (2.6-fold);<br>= tmax	72

SLNs: Solid Lipid nanoparticles; PVA: polyvinyl alcohol; PLGA: poly (dl-lactide-co-glycolide; AUC: area under the curve; MRT: mean residence time; SNEDDS: Self- Nanoemulsifying (Drug) Delivery Systems; HPβCD: 2-hydroxyl propy- beta-cyclodextrin; NP: nanoparticles; HPCD-NPZ: nanoparticles in the presence of HP-β-CD.: PC. Phospolipid complex. SD: Sprague-Dawley, Cur: curcumina, Cur-glucu: curcumin glucuronide, Cur-sulf: curcumin sulfate; TPGS: D-α-tocopheryl polyethylene glycol 1,000 succinate; CH-SLNs: chitosan-solid lipid nanoparticles; TMC-SLNs: trimethyl chitosan-solid lipid nanoparticles; D-Cur (demethoxycurcumin), MCT: medium chain triacylglycerols: PEG: polyethylene glycol; SD: Sprague-Dawley; TMC-g-PA: trimethyl chitosan conjugated with palmitic acid

1. Patil, S.; Choudhary, B.; Rathore, A.; Roy, K.; Mahadik, K. Enhanced oral bioavailability and anticancer activity of novel curcumin loaded mixed micelles in human lung cancer cells. *Phytomedicine* **2015,** *22*, 1103-1111.

2. Liu, J.; Lei, L.; Ye, F.; Zhou, Y.; Younis, H. G. R.; Zhao, G. Aggregates of octenylsuccinate oat β-glucan as novel capsules to stabilize curcumin over food processing, storage and digestive fluids and to enhance its bioavailability. *Food Funct.* **2018,** *9* , 491-501.

3. Peng, S.; Li, Z.; Zou, L.; Liu, W.; Liu, C.; McClements, D. J. Enhancement of curcumin bioavailability by encapsulation in sophorolipid-coated nanoparticles: an *in vitro* and *in vivo* study. *J. Agric. Food Chem.* **2018,** *66* , 1488-1497.

4. Liu, C.; Cheng, F.; Yang, X. Fabrication of a soybean bowman-birk inhibitor (BBI) nanodelivery carrier to improve bioavailability of curcumin. *J. Agric. Food Chem.* **2017,** *65* , 2426-2434.

5. Xie, X.; Tao, Q.; Zou, Y.; Zhang, F.; Guo, M.; Wang, Y.; Wang, H.; Zhou, Q.; Yu, S. PLGA nanoparticles improve the oral bioavailability of curcumin in rats: characterizations and mechanisms. *J. Agric. Food Chem.* **2011,** *59*, 9280-9289.

6. Khalil, N. M.; do Nascimento, T. C.; Casa, D. M.; Dalmolin, L. F.; de Mattos, A. C.; Hoss, I.; Romano, M. A.; Mainardes, R. M. Pharmacokinetics of curcumin-loaded PLGA and PLGA-PEG blend nanoparticles after oral administration in rats. *Colloids Surf. B: Biointerfaces* **2013,** *101*, 353-60.

7. Shaikh, J.; Ankola, D. D.; Beniwal, V.; Singh, D.; Kumar, M. N. V. R. Nanoparticle encapsulation improves oral bioavailability of curcumin by at least 9-fold when compared to curcumin administered with piperine as absorption enhancer. *Eur.J. Pharm. Sci.* **2009,** *37* , 223-230.

8. Li, N.; Wang, N.; Wu, T.; Qiu, C.; Wang, X.; Jiang, S.; Zhang, Z.; Liu, T.; Wei, C.; Wang, T. Preparation of curcumin-hydroxypropyl-*β*-cyclodextrin inclusion complex by cosolvency-lyophilization procedure to enhance oral bioavailability of the drug. *Drug Dev. Ind. Pharm.* **2018,** *44* , 1966-1974.

9. Xu, G.; Wang, C.; Yao, P. Stable emulsion produced from casein and soy polysaccharide compacted complex for protection and oral delivery of curcumin. *Food Hydrocoll.* **2017,** *71*, 108-117.

10. Vecchione, R.; Quagliariello, V.; Calabria, D.; Calcagno, V.; De Luca, E.; Iaffaioli, R. V.; Netti, P. A. Curcumin bioavailability from oil in water nano-emulsions: *In vitro* and *in vivo* study on the dimensional, compositional and interactional dependence. *J. Control. Release* **2016,** *233*, 88-100.

11. Cui, J.; Yu, B.; Zhao, Y.; Zhu, W.; Li, H.; Lou, H.; Zhai, G. Enhancement of oral absorption of curcumin by self-microemulsifying drug delivery systems. *Int. J. Pharm.* **2009,** *371*, 148-55.

12. Yu, H.; Huang, Q. Improving the oral bioavailability of curcumin using novel organogel-based nanoemulsions. *J. Agric. Food Chem.* **2012,** *60*, 5373-5379.

13. Maiti, K.; Mukherjee, K.; Gantait, A.; Saha, B. P.; Mukherjee, P. K. Curcumin-phospholipid complex: Preparation, therapeutic evaluation and pharmacokinetic study in rats. *Int. J. Pharm.* **2007,** *330*, 155-63.

14. Liu, A.; Lou, H.; Zhao, L.; Fan, P. Validated LC/MS/MS assay for curcumin and tetrahydrocurcumin in rat plasma and application to pharmacokinetic study of phospholipid complex of curcumin. *J. Pharm. Biomed. Anal.* **2006,** *40* , 720-727.

15. Marczylo, T. H.; Verschoyle, R. D.; Cooke, D. N.; Morazzoni, P.; Steward, W. P.; Gescher, A. J. Comparison of systemic availability of curcumin with that of curcumin formulated with phosphatidylcholine. *Cancer Chemother. Pharmacol.* **2007,** *60*, 171-177.

16. Takahashi, M.; Uechi, S.; Takara, K.; Asikin, Y.; Wada, K. Evaluation of an oral carrier system in rats: bioavailability and antioxidant properties of liposome-encapsulated curcumin. *J. Agric. Food Chem.* **2009,** *57*, 9141-9146.

17. Chen, H.; Wu, J.; Sun, M.; Guo, C.; Yu, A.; Cao, F.; Zhao, L.; Tan, Q.; Zhai, G. N-trimethyl chitosan chloride-coated liposomes for the oral delivery of curcumin. *J. Liposome Res.* **2012,** *22*, 100-109.

18. Kakkar, V.; Singh, S.; Singla, D.; Kaur, I. P. Exploring solid lipid nanoparticles to enhance the oral bioavailability of curcumin. *Mol. Nutr. Food Res.* **2011,** *55*, 495-503.

19. Ramalingam, P.; Ko, Y. T. Enhanced oral delivery of curcumin from N-trimethyl chitosan surface-modified solid lipid nanoparticles: pharmacokinetic and brain distribution evaluations. *Pharm. Res.* **2015,** *32*, 389-402.

20. Wang, Y.; Wang, C.; Zhao, J.; Ding, Y.; Li, L. A cost-effective method to prepare curcumin nanosuspensions with enhanced oral bioavailability. *J. Colloid Interface Sci.* **2017,** *485*, 91-98.

21. Dekate, S.; Bhairy, S.; Hirlekar, R. Preparation and characterization of oral nanosuspension loaded with curcumin. *Int. J. Pharm. Pharm.* **2018,** *10*, 90-95.

22. Gao, Y.; Wang, C.; Sun, M.; Wang, X.; Yu, A.; Li, A.; Zhai, G. In vivo evaluation of curcumin loaded nanosuspensions by oral administration. *J. Biomed. Nanotech.* **2012,** *8*, 659-668.

23. Munjal, B.; Pawar, Y. B.; Patel, S. B.; Bansal, A. K. Comparative oral bioavailability advantage from curcumin formulations. *Drug Deliv. Transl. Res.* **2011,** *1*, 322-331.

24. Khan, A. W.; Kotta, S.; Ansari, S. H.; Sharma, R. K.; Ali, J. Self-nanoemulsifying drug delivery system (SNEDDS) of the poorly water-soluble grapefruit flavonoid naringenin: design, characterization, *in vitro* and *in vivo* evaluation. *Drug Deliv.* **2015,** *22*, 552-61.

25. Wang, Y.; Wang, S.; Firempong, C. K.; Zhang, H.; Wang, M.; Zhang, Y.; Zhu, Y.; Yu, J.; Xu, X. Enhanced solubility and bioavailability of naringenin via liposomal nanoformulation: preparation and in vitro and in vivo evaluations. *AAPS PharmSciTech* **2017,** *18*, 586-594.

26. Kim, B. K.; Cho, A. R.; Park, D. J. Enhancing oral bioavailability using preparations of apigenin-loaded W/O/W emulsions: *In vitro* and *in vivo* evaluations. *Food Chem.* **2016,** *206*, 85-91.

27. Wei, Y.; Guo, J.; Zheng, X.; Wu, J.; Zhou, Y.; Yu, Y.; Ye, Y.; Zhang, L.; Zhao, L. Preparation, pharmacokinetics and biodistribution of baicalin-loaded liposomes. *Int. J. Nanomedicine* **2014,** *9*, 3623-3630.

28. Hao, J.; Wang, F.; Wang, X.; Zhang, D.; Bi, Y.; Gao, Y.; Zhao, X.; Zhang, Q. Development and optimization of baicalin-loaded solid lipid nanoparticles prepared by coacervation method using central composite design. *Eur. J. Pharm. Sci.* **2012,** *47*, 497-505.

29. Zhao, L.; Wei, Y.; Huang, Y.; He, B.; Zhou, Y.; Fu, J. Nanoemulsion improves the oral bioavailability of baicalin in rats: in vitro and in vivo evaluation. *Int. J. Nanomedicine* **2013,** *8*, 3769-3779.

30. Wu, L.; Bi, Y.; Wu, H. Formulation optimization and the absorption mechanisms of nanoemulsion in improving baicalin oral exposure. *Drug Dev. Ind. Pharm.* **2018,** *44* , 266-275.

31. Zhang, H.; Yang, X.; Zhao, L.; Jiao, Y.; Liu, J.; Zhai, G. In vitro and in vivo study of baicalin-loaded mixed micelles for oral delivery. *Drug Deliv.* **2016,** *23*, 1933-1939.

32. Li, J.; Jiang, Q.; Deng, P.; Chen, Q.; Yu, M.; Shang, J.; Li, W. The formation of a host-guest inclusion complex system between *β*-cyclodextrin and baicalin and its dissolution characteristics. *J. Pharm. Pharmacol.* **2017,** *69*, 663-674.

33. Li, B.; He, M.; Li, W.; Luo, Z.; Guo, Y.; Li, Y.; Zang, C.; Wang, B.; Li, F.; Li, S.; Ji, P. Dissolution and pharmacokinetics of baicalin-polyvinylpyrrolidone coprecipitate. *J. Pharm. Pharmacol.* **2013,** *65*, 1670-1678.

34. Wu, H.; Long, X.; Yuan, F.; Chen, L.; Pan, S.; Liu, Y.; Stowell, Y.; Li, X. Combined use of phospholipid complexes and self-emulsifying microemulsions for improving the oral absorption of a BCS class IV compound, baicalin. *Acta Pharm. Sin. B.* **2014,** *4*, 217-226.

35. Kadari, A.; Gudem, S.; Kulhari, H.; Bhandi, M. M.; Borkar, R. M.; Kolapalli, V. R.; Sistla, R. Enhanced oral bioavailability and anticancer efficacy of fisetin by encapsulating as inclusion complex with HPβCD in polymeric nanoparticles. *Drug Deliv.* **2017,** *24*, 224-232.

36. Li, H.; Zhao, X.; Ma, Y.; Zhai, G.; Li, L.; Lou, H. Enhancement of gastrointestinal absorption of quercetin by solid lipid nanoparticles. *J. Control Release* **2009,** *133*, 238-44.

37. Sun, M.; Gao, Y.; Pei, Y.; Guo, C.; Li, H.; Cao, F.; Yu, A.; Zhai, G. Development of nanosuspension formulation for oral delivery of quercetin. *J. Biomed. nanotech.* **2010,** *6*, 325-32.

38. Li, W.; Yi, S.; Wang, Z.; Chen, S.; Xin, S.; Xie, J.; Zhao, C. Self-nanoemulsifying drug delivery system of persimmon leaf extract: Optimization and bioavailability studies. *Int. J. Pharm.* **2011,** *420*, 161-171.

39. Tran, T. H.; Guo, Y.; Song, D.; Bruno, R. S.; Lu, X. Quercetin-containing self-nanoemulsifying drug delivery system for improving oral bioavailability. *J. Pharma. Sci.* **2014,** *103* , 840-852.

40. Jain, S.; Jain, A. K.; Pohekar, M.; Thanki, K. Novel self-emulsifying formulation of quercetin for improved in vivo antioxidant potential: Implications for drug-induced cardiotoxicity and nephrotoxicity. *Free Radic. Biol. Med.* **2013,** *65*, 117-130.

41. Jain, A. K.; Thanki, K.; Jain, S. Co-encapsulation of tamoxifen and quercetin in polymeric nanoparticles: implications on oral bioavailability, antitumor efficacy, and drug-induced toxicity. *Mol. Pharm.* **2013,** *10*, 3459-3474.

42. Dian, L.; Yu, E.; Chen, X.; Wen, X.; Zhang, Z.; Qin, L.; Wang, Q.; Li, G.; Wu, C. Enhancing oral bioavailability of quercetin using novel soluplus polymeric micelles. *Nanoscale Res. Lett.* **2014,** *9*, 2406.

43. Liu, L.; Tang, Y.; Gao, C.; Li, Y.; Chen, S.; Xiong, T.; Li, J.; Du, M.; Gong, Z.; Chen, H.; Liu, L.; Yao, P. Characterization and biodistribution *in vivo* of quercetin-loaded cationic nanostructured lipid carriers.*Colloids Surf. B: Biointerfaces.* **2014,** *115*, 125-31.

44. Penalva, R.; González-Navarro, C. J.; Gamazo, C.; Esparza, I.; Irache, J. M. Zein nanoparticles for oral delivery of quercetin: pharmacokinetic studies and preventive anti-inflammatory effects in a mouse model of endotoxemia. *Nanomed.: Nanotechnol. Biol. Med.* **2017,** *13*, 103-110.

45. Peñalva, R.; Esparza, I.; Morales-Gracia, J.; González-Navarro, C. J.; Larrañeta, E.; Irache, J. M. Casein nanoparticles in combination with 2-hydroxypropyl-β-cyclodextrin improves the oral bioavailability of quercetin. *Int. J. Pharm.* **2019,** *570*, 118652.

46. Lee, S.-H.; Kim, Y. H.; Yu, H.-J.; Cho, N.-S.; Kim, T.-H.; Kim, D.-C.; Chung, C.-B.; Hwang, Y.-I.; Kim, K. H. Enhanced bioavailability of soy isoflavones by complexation with β-cyclodextrin in rats. *Biosci. Biotechnol. Biochem.***2007,** *71*, 2927-2933.

47. Shen, Q.; Li, X.; Yuan, D.; Jia, W. Enhanced oral bioavailability of daidzein by self-microemulsifying drug delivery system. *Chem. Pharm. Bull.* **2010,** *58*, 639-43.

48. Zhang, Z.; Huang, Y.; Gao, F.; Bu, H.; Gu, W.; Li, Y. Daidzein-phospholipid complex loaded lipid nanocarriers improved oral absorption: *in vitro* characteristics and *in vivo* behavior in rats. *Nanoscale* **2011,** *3*, 1780-1787.

49. Cohen, R.; Schwartz, B.; Peri, I.; Shimoni, E. Improving bioavailability and stability of genistein by complexation with high-amylose corn starch. *J. Agric. Food Chem.* **2011,** *59*, 7932-7938.

50. Shen, H.; He, D.; Wang, S.; Ding, P.; Wang, J.; Ju, J. Preparation, characterization, and pharmacokinetics study of a novel genistein-loaded mixed micelles system. *Drug. Dev. Ind. Pharm.* **2018,** *44*, 1536-1542.

51. Luo, C. F.; Yuan, M.; Chen, M. S.; Liu, S. M.; Zhu, L.; Huang, B. Y.; Liu, X. W.; Xiong, W. Pharmacokinetics, tissue distribution and relative bioavailability of puerarin solid lipid nanoparticles following oral administration. *Int. J. Pharm.* **2011,** *410*, 138-44.

52. Smith, A.; Giunta, B.; Bickford, P. C.; Fountain, M.; Tan, J.; Shytle, R. D. Nanolipidic particles improve the bioavailability and alpha-secretase inducing ability of epigallocatechin-3-gallate (EGCG) for the treatment of Alzheimer's disease. *Int. J. Pharm.* **2010,** *389*, 207-12.

53. Dube, A.; Nicolazzo, J. A.; Larson, I. Chitosan nanoparticles enhance the plasma exposure of (-)-epigallocatechin gallate in mice through an enhancement in intestinal stability. *Eur. J. Pharm. Sci.***2011,** *44*, 422-426.

54. Huang, Y. B.; Tsai, M. J.; Wu, P. C.; Tsai, Y. H.; Wu, Y. H.; Fang, J. Y. Elastic liposomes as carriers for oral delivery and the brain distribution of (+)-catechin. *J. Drug Target.* **2011,** *19*, 709-718.

55. Ezzat, H. M.; Elnaggar, Y. S. R.; Abdallah, O. Y. Improved oral bioavailability of the anticancer drug catechin using chitosomes: Design, in-vitro appraisal and *in-vivo* studies. *Int. J. Pharm.* **2019,** *565*, 488-498.

56. Singh, G.; Pai, R. S. Enhanced oral bioavailability of (+)-catechin by a self double-emulsifying drug delivery system (SDEDDS): A new platform for oral delivery of biopharmaceutics classification system class III drugs. *Nanomedicine and Nanobiology* **2014,** *1* , 51-56.

57. Peng, Y.; Meng, Q.; Zhou, J.; Chen, B.; Xi, J.; Long, P.; Zhang, L.; Hou, R. Nanoemulsion delivery system of tea polyphenols enhanced the bioavailability of catechins in rats. *Food Chem.* **2018,** *242*, 527-532.

58. Augustin, M. A.; Abeywardena, M. Y.; Patten, G.; Head, R.; Lockett, T.; De Luca, A.; Sanguansri, L. Effects of microencapsulation on the gastrointestinal transit and tissue distribution of a bioactive mixture of fish oil, tributyrin and resveratrol. *J.Funct. Foods* **2011,** *3*, 25-37.

59. Singh, G.; Pai, R. S. *In-vitro/in-vivo* characterization of trans-resveratrol-loaded nanoparticulate drug delivery system for oral administration. *J. Pharm. Pharmacol.* **2014,** *66* , 1062-1076.

60. Singh, G.; Pai, R. S. Optimized PLGA nanoparticle platform for orally dosed trans-resveratrol with enhanced bioavailability potential. *Expert Opin. Drug Deliv.* **2014,** *11*, 647-659.

61. Penalva, R.; Esparza, I.; Larraneta, E.; González-Navarro, C. J.; Gamazo, C.; Irache, J. M. Zein-based nanoparticles improve the oral bioavailability of resveratrol and its anti-inflammatory effects in a mouse model of endotoxic shock. *J. Agric. Food Chem.* **2015,** *63* , 5603-5611.

62. Peñalva, R.; Morales, J.; González-Navarro, C. J.; Larrañeta, E.; Quincoces, G.; Peñuelas, I.; Irache, J. M. Increased oral bioavailability of resveratrol by its encapsulation in casein nanoparticles. *Int. J. Mol. Sci.* **2018,** *19*,2816.

63. Zu, Y.; Zhang, Y.; Wang, W.; Zhao, X.; Han, X.; Wang, K.; Ge, Y. Preparation and *in vitro/in vivo* evaluation of resveratrol-loaded carboxymethyl chitosan nanoparticles. *Drug Deliv.* **2016,** *23*, 981-991.

64. Pandita, D.; Kumar, S.; Poonia, N.; Lather, V. Solid lipid nanoparticles enhance oral bioavailability of resveratrol, a natural polyphenol. *Food Res. Int.* **2014,** *62*, 1165-1174.

65. Ramalingam, P.; Ko, Y. T. Improved oral delivery of resveratrol from N-trimethyl chitosan-g-palmitic acid surface-modified solid lipid nanoparticles. *Colloids Surf. B: Biointerfaces* **2016,** *139*, 52-61.

66. Singh, G.; Pai, R. S. Trans-resveratrol self-nano-emulsifying drug delivery system (SNEDDS) with enhanced bioavailability potential: optimization, pharmacokinetics and in situ single pass intestinal perfusion (SPIP) studies. *Drug Deliv.* **2015,** *22*, 522-530.

67. Jangid, A. K.; Patel, K.; Jain, P.; Patel, S.; Medicherla, K.; Pooja, D.; Kulhari, H. Carrier-free resveratrol nanoparticles: formulation development, in-vitro anticancer activity, and oral bioavailability evaluation. *Mater. Lett.* **2021,** *302*, 130340.

68. Nallamuthu, I.; Devi, A.; Khanum, F. Chlorogenic acid loaded chitosan nanoparticles with sustained release property, retained antioxidant activity and enhanced bioavailability. *Asian J. Pharm. Sci.* **2015,** *10*, 203-211.

69. Panwar, R.; Raghuwanshi, N.; Srivastava, A. K.; Sharma, A. K.; Pruthi, V. *In-vivo* sustained release of nanoencapsulated ferulic acid and its impact in induced diabetes. *Mater. Sci. Eng. C.* **2018,** *92*, 381-392.

70. Liu, Y.; Sun, C.; Li, W.; Adu-Frimpong, M.; Wang, Q.; Yu, J.; Xu, X. Preparation and characterization of syringic acid-loaded TPGS liposome with enhanced oral bioavailability and in vivo antioxidant efficiency. *AAPS PharmSciTech* **2019,** *20*, 98.

71. Wang, S.-T.; Chou, C.-T.; Su, N.-W. A food-grade self-nanoemulsifying delivery system for enhancing oral bioavailability of ellagic acid. *J. Funct. Foods* **2017,** *34*, 207-215.

72. Murugan, V.; Mukherjee, K.; Maiti, K.; Mukherjee, P. K. Enhanced oral bioavailability and antioxidant profile of ellagic acid by phospholipids. *J.Agric. Food Chem.* **2009,** *57* (11), 4559-4565.
